# Submerged Eutectic-Assisted, Solvent-Free Mechanochemical Formation of a Propranolol Salt and Its Other Multicomponent Solids

**DOI:** 10.3390/pharmaceutics13122125

**Published:** 2021-12-09

**Authors:** Klaudia Bialek, Zaneta Wojnarowska, Marcin Skotnicki, Brendan Twamley, Marian Paluch, Lidia Tajber

**Affiliations:** 1 School of Pharmacy and Pharmaceutical Sciences, Trinity College Dublin, D02 PN40 Dublin, Ireland; bialekk@tcd.ie (K.B.); zaneta.wojnarowska@smcebi.edu.pl (Z.W.); 2 Institute of Physics, University of Silesia, SMCEBI, 75 Pulku Piechoty 1A, 41-500 Chorzow, Poland; marian.paluch@us.edu.pl; 3 Chair and Department of Pharmaceutical Technology, Poznan University of Medical Sciences, ul. Grunwaldzka 6, 60-780 Poznan, Poland; marcskot@ump.edu.pl; 4 School of Chemistry, Trinity College Dublin, D02 PN40 Dublin, Ireland; twamleyb@tcd.ie

**Keywords:** propranolol, capric acid, salt, eutectic, submerged eutectic, mechanochemistry, solid state

## Abstract

Salt preparation via a solid-state reaction offers a solution to challenges posed by current pharmaceutical research, which include combining development of novel forms of active pharmaceutical ingredients with greener, sustainable synthesis. This work investigated in detail the mechanism of salt formation between propranolol (PRO) and capric acid (CAP) and explored the solid eutectic phases comprising this salt, propranolol caprate (PRC). The salt structure was solved by X-ray diffraction, and the properties in the crystalline and supercooled states were fully characterised using thermal analysis, nuclear magnetic resonance, Fourier-transform infrared spectroscopy and broadband dielectric spectroscopy (BDS). PRC forms via a submerged eutectic phase composed of PRO and CAP, below room temperature, by mechanochemistry without an extra input of energy. Two other solid eutectic phases are composed of PRC and either CAP or PRO, at 0.28 and 0.82 mol fraction of PRO, respectively. BDS indicated that the supercooled PRC has ionic character, whereas the supercooled PRC-PRO eutectic had predominantly non-ionic properties despite comprising the salt. In conclusion, knowledge of the mechanism of formation of multicomponent systems can help in designing more sustainable pharmaceutical processes.

## 1. Introduction

**Figure 1:**
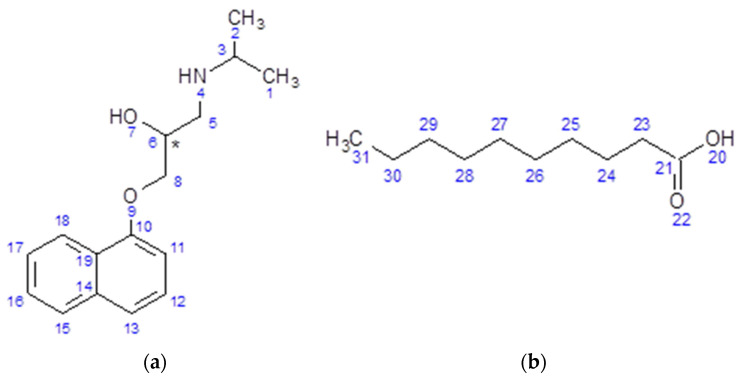
Chemical structures of (**a**) propranolol (PRO), (Mr = 259.34 g/mol, pKa = 9.7) and (**b**) capric acid (CAP), (Mr = 172.27 g/mol, pKa = 4.9)) with the carbon, nitrogen and oxygen atoms numbered.

Propranolol (PRO, Figure 1) is a first-generation nonselective β-blocker, blocking both β1-adrenergic and β2-adrenergic receptors [1]. It is primarily used in the treatment of cardiovascular conditions such as hypertension, cardiac arrhythmias and angina pectoris [2]. It has recently been approved as a first line therapy drug for treatment of infantile haemangioma (IH) [3]. IH is the most common benign vascular tumour, affecting around 5% of the infantile population [4]. Approximately 10% of all diagnosed IH cases are life-threatening and require medical intervention [5]. PRO, due to its low solubility (609 mg/L) [6] has been marketed as a hydrochloride salt (solubility of 97.9 g/L) [7], and it is available in a range of oral formulations, such as immediate- and extended-release tablets as well as an oral solution. In the context of IH, even though the PRO·HCl oral solution is effective in the treatment of this ailment, the salt is poorly bioavailable (13–23%) and shows extensive hepatic first pass metabolism [8]. Furthermore, oral administration is impractical in the treatment of paediatric patients, and poor palatability of the formulation may lead to insufficient patient compliance and therefore inconsistency of dosing [9,10]. In contrast to oral formulations, the topical route is convenient and produces a minimal impact on the lifestyle of the infant [11]. However, only 10–37% of the applied PRO·HCl permeates through the skin [12]; therefore, there is a need to improve the poor skin passage of PRO.

The strategy of changing the counterion of PRO could be effectively used to increase permeation of PRO through the skin. Attempts have been made to develop more transdermally permeable forms of PRO. Monocarboxylic acids (C6 to C18 fatty acids) as potential penetration enhancers for the transdermal transport of PRO were studied by Stott et al. [13]. The use of fatty acids is a common strategy to increase skin permeability [14]. The study undertaken by Stott et al. concluded that the binary PRO:fatty acid addition compounds formed salts at a 1:1 molar ratio and were found to permeate skin via an ion-pair mechanism. Interestingly, the above study also presented data on thermal analysis of binary mixtures of PRO with capric (C10) and lauric (C12) acids. The phase diagrams constructed for these combinations showed the formation of eutectic systems; however, not only one, but two eutectic phases, on either side of the PRO:acid 1:1 compositions, were identified. It has been identified that the formation of a eutectic between the API and a fatty acid may further improve PRO skin passage [15].

Mechanochemical cocrystal and salt synthesis has recently been investigated by many research groups as a more sustainable route. In contrast to classical solvent-base crystallisation, mechanochemical synthesis does not involve the use of solvent. This approach is more environmentally friendly and allows the production of solvent-sensitive reactants and products [16,17]. Furthermore, the mechanochemical approach can be employed to discover other solid phases (also salts) unstable in solvents [18,19]. Trask and co-workers proved that the solvent-free preparation of samples can be used to produce new crystal forms of trimethoprim and pyrimethamine, which were not observed to form from solution [20]. To date, little attention has been paid to salt formation mechanisms through mechanochemistry. To the best of our knowledge, only three mechanisms of mechanochemical salt formation have been described. The first mechanism involves salt formation achieved by amorphisation of the reactants. Paluch and co-workers successfully obtained ciprofloxacin succinate salt in a 2:1 stoichiometry by ball milling [19], and later Mesallati et al. produced a range of salts of ciprofloxacin and amino acids by the same method [21]. A salt between glycine and malonic acid has successfully been formed by milling, impact and shear treatment [22]. The other two mechanochemical routes involve a liquid intermediate. In the case of the second mechanism, the liquid intermediate is induced by heating of the reactants. Lee et al. reported on the haloperidol−maleic acid salt synthesised with the use of a twin-screw hot melt extruder [23]. During the extrusion process, the liquid intermediate was formed when reactants were heated. The last mechanism of salt synthesis involves formation of a submerged eutectic. An idea of the submerged eutectic forming between the reactants and acting as a reaction intermediate to form a co-crystal has previously been described by Chadwick et al. [16]. Recently, a similar concept of a submerged eutectic (also referred to as ‘deep eutectic’) facilitating salt/ionic liquid formation was proposed by Zotova et al. [24].

We previously investigated a binary system of PRO and sebacic acid [25]. Sebacic acid is the equivalent dicarboxylic acid of capric acid (CAP), and its structure is presented in Figure 1. The studies showed that PRO was able to form a salt with sebacic acid (SEBA) in a 2:1 ratio. The salt further interacts with SEBA to form a eutectic phase. It was found that the salt, dipropranolol sebacate, can be obtained by different methods such as conventional solvent crystallisation, heat-induced crystallisation and interestingly, mechanochemically by grinding. Despite the early work of Stott et al. [13] on the mixtures of PRO and CAP, many questions remain to be answered about the identity of the multicomponent forms, intermolecular interactions and the formation of eutectic phases. Therefore, the aim of this work was to investigate the nature of bonding between PRO and CAP using solution and solid-state NMR as well as broadband dielectric spectroscopy. Additionally, the structure of the complex was ultimately resolved, for the first time, by X-ray studies. This paper also examined the possibility of the PRO-CAP salt formation through mechanochemistry and compared the outcomes to those generated by Stott et al., who used a solvent for sample preparation. Finally, the eutectic phases were characterised in-depth, and key physiochemical characteristics were determined.

## 2. Materials and Methods

### 2.1. Materials

Propranolol hydrochloride (PRO·HCl) was purchased from Tokyo Chemical Industry (Tokyo, Japan). Capric acid (CAP) was obtained from Sigma-Aldrich Chemie GmbH, (Steinheim, Germany). Ethanol (HPLC grade) was purchased from Fisher Scientific (Loughborough, UK). All other chemicals and solvents used were of analytical grade.

### 2.2. Methods

#### 2.2.1. Sample Preparation

##### PRO Free Base Synthesis

The PRO base was obtained from the PRO·HCl salt as previously described by Bialek et al. [25]. Briefly, a quantity of 1 g of PRO·HCl was dissolved in 50 mL of deionised water. Saturated solution of NaHCO_3_ was added until pH of 9 was reached. PRO base precipitated as a white solid. The suspension was then separated by filtration under vacuum using a ceramic Buchner filter equipped with a 0.45 µm polypropylene membrane disc filter. The recovered solid was washed with 50 mL of deionised water to eliminate any remaining residue of the hydrochloride salt. PRO base was then oven-dried (Memmert UL 40, Schwabach, Germany) at 60 °C overnight.

##### Synthesis of Propranolol Caprate (PRC) Salt

To obtain the propranolol caprate salt (PRC), PRO and CAP were dissolved in hot ethanol in a 1:1 molar ratio. The reaction solution was heated to 80 °C while constantly stirring using a magnetic stirrer (Stuart SD162 Hotplate stirrer, Stone, UK) to ensure complete dissolution of the powders. The solution was then cooled rapidly by placing the reaction vessel on an ice bath and then kept at 4 °C overnight to allow salt precipitation. The precipitated PRC was filtered under vacuum using a ceramic Buchner filter equipped with a 0.45 µm polypropylene membrane disc filter and washed with ice-cold ethanol. PRC was dried overnight (Memmert UL 40) at 60 °C. The salt was stored in a sealed glass vial at ambient temperature and moisture.

##### Preparation of PRO-CAP, PRC-CAP and PRC-PRO Physical Mixtures

Physical mixtures of PRO-CAP, PRC-CAP and PRC-PRO were prepared in approx. 50 mg aliquots, by co-grinding PRO-CAP, PRC-CAP and PRC-PRO at appropriate molar ratios in an agate mortar and pestle to obtain homogenous mixtures. PRO-CAP samples ranging from 0.1 to 0.67 mol of PRO and all PRC-CAP samples were prepared at 4 °C, whereas PRO-CAP samples ranging from 0.7 to 0.9 mol of PRO and all PRC-PRO samples were prepared at ambient temperature.

##### Preparation of Samples for Contact and Grinding-Assisted Solid-State Reaction

Firstly, PRO was pre-dried in the Memmer UL 40 oven at 70 °C for 1 h. CAP was used as received. For the contact solid-state reaction, 0.8 mol of CAP and 0.2 mol of pre-dried PRO were mixed gently in a 20 mL glass vial with a spatula at 4 °C to obtain approximately 200 mg sample. For the grinding-assisted solid-state reaction, the same quantities of CAP and PRO were ground in an agate pestle and mortar at 4 °C. The sample was then transferred to a 20 mL glass vial. The samples were hermetically sealed and stored at 4 °C until analysed.

#### 2.2.2. Single Crystal X-ray Diffraction (SCXRD)

Data for PRC were collected on a Bruker APEX Kappa DUO (Billerica, MA, USA) using Cu Kα radiation (λ = 1.54178 Å). The sample was mounted on a MiTeGen cryoloop (Ithaca, NY, USA), and data collected at 180(2) K using an Oxford Cobra cryosystem (Oxford, UK). Bruker APEX software (Billerica, MA, USA) was used to collect, reduce data and determine the space group. Absorption corrections were applied using SADABS. The structure was solved using the XT structure solution program [26] using intrinsic phasing and refined with the XL refinement package [27] using least squares minimization with OLEX2 [28]. All non-hydrogen atoms were refined anisotropically. Hydrogen atoms were assigned to calculated positions using a riding model with appropriately fixed isotropic thermal parameters. Molecular graphics were generated using OLEX2. The aliphatic chain on one capric acid was disordered and modelled over two locations with 73:27% occupancy. Restraints were used to model the low occupancy moiety (DFIX, SADI, ISOR). Crystallographic data for the structure in this paper have been deposited with the Cambridge Crystallographic Data Centre as supplementary publication no. 2046913. Copies of the data can be obtained, free of charge, on application to CCDC, 12 Union Road, Cambridge CB2 1EZ, UK, (fax: +44-(0)1223-336033 or e-mail: deposit@ccdc.cam.ac.uk).

#### 2.2.3. Fourier Transform Infrared Spectroscopy (FTIR)

FTIR of solid samples was carried out using a Spectrum One FT-IR spectrometer (PerkinElmer, Shelton, CT, USA) equipped with Universal ATR Sampling Accessory. A spectral range of 650−4000 cm^−1^, resolution of 4 cm^−1^ and accumulation of 64 scans were used. The Spectrum Software version 10.6.0 was used for spectral analysis, which involved interactive baseline correction and normalisation of spectral intensities.

#### 2.2.4. Solution and Solid-State Nuclear Magnetic Resonance (NMR)

Solution NMR experiments were carried out on Bruker Avance III 400 and Bruker Avance II 600 machines (Billerica, MA, USA) with ^1^H and ^13^C frequencies of 400.23, 100.64 and 600.13, 150.90 MHz, respectively. Samples were dissolved (approx. 5 mg) in 10 mL of DMSO-*d_6_*. All measurements were performed at 25 °C.

Carbon-13 solid-state NMR spectra were recorded with cross-polarisation (CP) and magic-angle spinning (MAS) using Bruker Avance HD 400 NMR (Billerica, MA, USA) and Agilent 800 DD2 (Santa Clara, CA, USA) spectrometers, operating at ^13^C frequencies of 100.61 and 201.12 MHz, respectively. A probe using 3.2 mm diameter rotor made of zirconia was employed. Typical operating conditions used a CP contact time of 1 ms, a recycle delay of 4 s, 64 to 1024 transients, and spinning rate of 20 kHz. The ^1^H−^13^C heteronuclear correlations (HETCOR) were obtained as described by Van Rossum et al. [29] using the Bruker Avance HD 400 NMR spectrometer employing 3.2 mm rotor with frequency switched Lee−Goldburg CP, two-pulse phase modulation decoupling, contact time of 0.1 ms, 48 transients, recycle delay of 4 s, and a spinning rate of 20 kHz. Carbon chemical shifts were referenced to the signal for tetramethylsilane via a replacement sample of solid adamantane (*δ_C_* = 38.4 ppm for the high-frequency line). All measurements were performed at 20 °C.

NMR data were processed using Gsim software [30] and ACD/Spectrus Processor (Advanced Chemistry Development Inc., Toronto, ON, Canada).

#### 2.2.5. Differential Scanning Calorimetry (DSC)

DSC measurements were carried out using a PerkinElmer Diamond DSC unit (Waltham, MA, USA) equipped with a ULSP B.V. 130 cooling system (Ede, Netherlands) [25]. Nitrogen (40 mL/min) was used as the purge gas. The DSC unit was calibrated using indium. Approximately 5−10 mg samples were accurately weighed and analysed in sealed 40 μL aluminium pans. The samples were held at either 25 °C or 4 °C (depending on sample preparation temperature, refer to Section 2.2.1) for 2 min in the DSC unit, then heated at a rate of 10 °C/min to 120 °C (first heating). The samples were then quench-cooled at a nominal rate of 300 °C/min to −60 °C, held at −60 °C for 5 min, and reheated at a rate of 10 °C to 120 °C (second heating). Pyris software 9.01.0174 was used to analyse the data [31].

#### 2.2.6. Thermogravimetry (TGA)

TGA was conducted on selected samples using a Mettler TG 50 module connected to a Mettler MT5 balance (Schwerzenbach, Switzerland). Samples, weighing approximately 8–10 mg, were placed in open aluminium pans and heated, in an inert atmosphere of nitrogen, up to 120 °C.

#### 2.2.7. Polarised Light Microscopy

The polarised light microscopy experiment was performed using an Olympus BX53 polarising optical microscope (Tokyo, Japan) equipped with a U-POT cross polarizer [24]. The microscope was equipped with Q IMAGING Fast 1394 camera (Olympus, Tokyo, Japan).

#### 2.2.8. Powder X-ray Diffraction (PXRD)

PXRD analysis was conducted using a Rigaku MiniFlex II Desktop X-ray diffractometer (Tokyo, Japan) equipped with Haskris cooling unit (Elmhurst, IL, USA). The unit operated at 30 kV and 15 mA with the Cu tube (1 kW normal focus) used as radiation source. The samples were analysed from 2 to 40° on the 2θ scale at a step size of 0.05° per second at room temperature. The samples were supported by a zero-background silicon sample holder (Rigaku, Tokyo, Japan) during analysis [25].

#### 2.2.9. Broadband Dielectric Spectroscopy (BDS)

The dielectric spectra of examined samples were measured using a Novocontrol Alpha Analyzer (Montabaur, Germany) as described previously [32]. The temperature was precisely controlled with a Quatro temperature controller using a nitrogen gas cryostat (accuracy better than 0.1 °C). During the measurements, the tested samples were placed between the steel electrodes of a capacitor (15 mm diameter) with a fixed distance between the electrodes (0.1 mm) provided by quartz ring. The applied electric field was 0.1 V.

## 3. Results

### 3.1. Characterisation of Propranolol Caprate Salt

**Figure 2:**
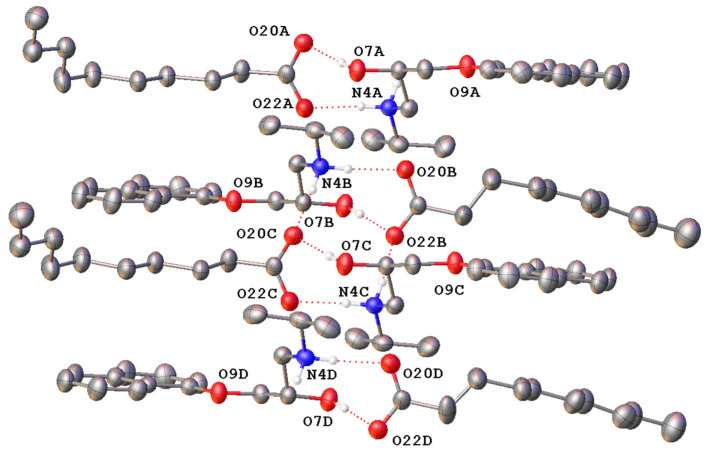
Molecular structure of the asymmetric unit of propranolol caprate (PRC) salt showing the four unique ion pairs with hydrogen bonding represented by dotted lines (only hydrogen atoms involved in hydrogen bonding shown). Only the majority occupied disordered caprate chain is shown (73% occupied). Heteroatoms labelled only, and atomic displacement shown at 50% probability.

The study performed by Stott et al. (2001) suggested that PRO and CAP can form an addition compound of an ionised nature (salt) at a 1:1 molar ratio; however, this salt was not isolated by this group. We made a successful attempt to determine the structure of this form, propranolol caprate (PRC), grown from an ethanolic solution, by single-crystal X-ray diffraction (SCXRD). The structure is shown in Figure 2. The molecule crystallises in the centrosymmetric space group P-1, indicating the salt is racemic. The asymmetric unit contains four unique ion pairs of PRO and CAP, indicating the complete molecule is formed in the ratio of 1:1 ratio between PRO and CAP. There is disorder in the aliphatic chain on one molecule of CAP (Figure S1). In the major and minor disordered moiety, each PRO cation is hydrogen-bonded to the CAP anion via the protonated amine as well as the hydroxyl group. Table S1 presents hydrogen bond lengths. A calculated PXRD pattern, generated from the data above, is shown overlaid on the experimental PXRD in Figure S2. The fit is visually reasonable and a Le Bail refinement yields R_p_ = 6.254 and R_wp_ = 8.587%.

**Figure 3:**
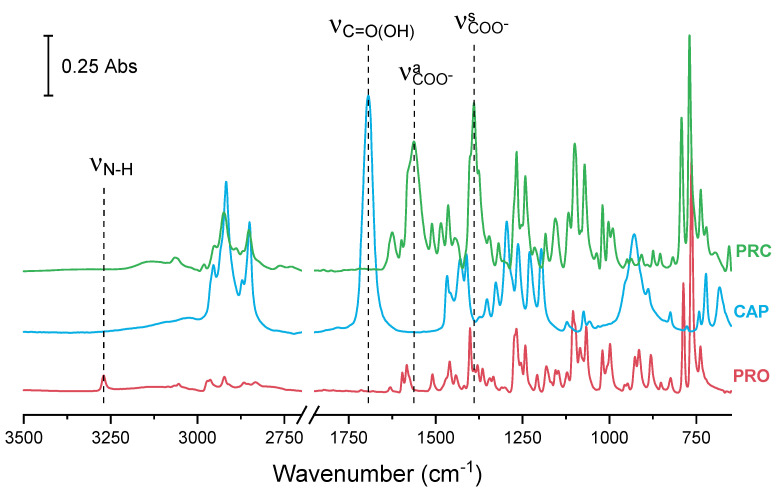
FTIR spectra of PRO, CAP and PRC. ν—stretching vibration, ν^s^—symmetric vibration, ν^a^—asymmetric vibration.

In addition to the structural analysis, characterisation of the salt in bulk was carried out by infrared spectroscopy (Figure 3). Infrared analysis showed that for pure PRO, a secondary amine (N–H) stretching band appeared as a weak peak at 3270 cm^−1^. Pure CAP showed a carbonyl (C=O) stretching vibration at 1694 cm^−1^. Typically, carbonyl peaks can be found in the 1740–1700 cm^−1^ region; however, the carbonyl of CAP appeared at a lower wavenumber as carboxylic acids including CAP form dimers. The hydrogen bonding in dimers lowers the frequency of the carbonyl stretching. In the spectrum of salt, ionisation of the carbonyl group was visible. The carbonyl stretching vibration disappeared and instead, two peaks of the symmetric and asymmetric vibration of the carboxylate ion appeared at 1390 and 1563 cm^−1^, respectively (Figure 3). This observation is in agreement with the SCRXD data, where ΔD C–O is less than 0.07 Å in all carbonyls in the caprate due to ionisation of the carbonyl group. There was additional evidence of interactions between the carbonyl of the acid (CAP) and the amine group of the base (PRO), as the peak for amine stretching had significantly decreased in intensity.

**Figure 4:**
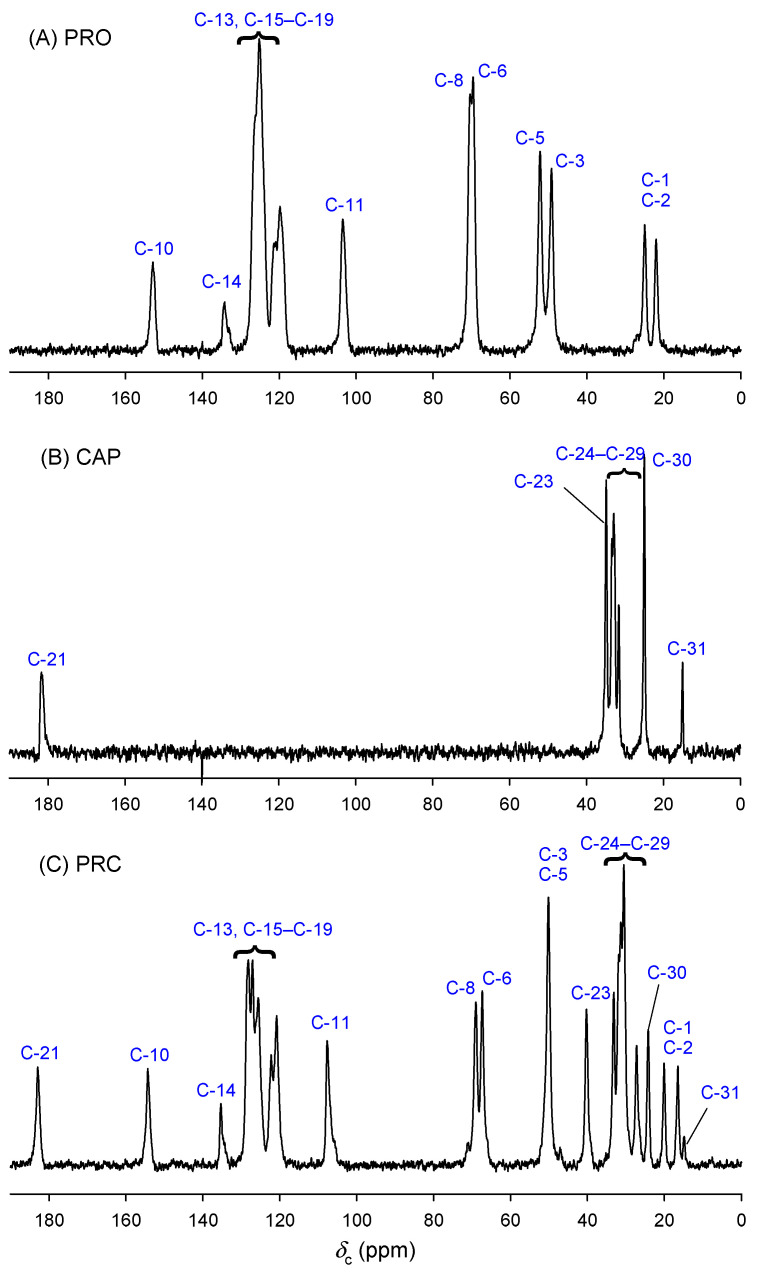
Carbon-13 CP MAS NMR spectra (201.12 MHz) of (**A**) PRO, (**B**) CAP and (**C**) PRC.

Solid-state NMR is a powerful tool for obtaining structural information on solid pharmaceuticals [33,34]. Figure 4 shows the ^13^C CP MAS NMR spectra of PRO, CAP and PRC. Standard NMR techniques (COSY, HSQC-DEPT and HMBC) were used to assign the solution-state spectra for PRO and PRC. Assignment of CAP was based on the previous reports [35,36]. Data for ^1^H solution-state NMR are provided in Tables S2–S4. The solid-state spectrum was assigned using solution-state NMR data and spectral editing techniques, i.e., depolarisation experiments with different inversion times and 2D HETCOR experiments (Figures S3–S6). The resulting assignments are presented in Table S2 and are in good agreement with ^13^C SSNMR data for the related compound propranolol oleate [37]. The solid-state spectra assignments contain ambiguities due to their lower resolutions (aliphatic region of CAP and aromatic region of PRO) compared with the solution-state spectra.

To confirm the salt formation between PRO and CAP, carbons bonded to amine group (C-3 and C-5) and carbons bonded to hydroxy (C-6), naphthyloxy groups (C-8) and carboxylic carbon (C-21) were investigated. Carbons C-5 and C-6 were found to be characteristic for bases and salts of beta-blockers investigated by solution-state NMR [38,39]. However, our solution-state NMR studies did not find significant differences between C-5 nitrogen-bonded and C-6 oxygen-bonded carbons for PRO (*δ*_C-5_ = 50.05 ppm, *δ*_C-6_ = 68.48 ppm) and PRC (*δ*_C-5_ = 50.07 ppm, *δ*_C-6_ = 68.41 ppm). A different situation was found in the solid state. Significant differences in chemical shifts were observed for amine group-bonded carbons (C-3 and C-5), i.e., 48.9, 51.8 ppm for PRO and only one overlapped the signal at 49.8 ppm for PRC. Moreover, oxygen-bonded carbons (C-6) also showed significant differences in chemical shifts, i.e., 69.2, and 67.1, for PRO and PRC, respectively. The upfield position of chemical shift in comparison to PRO was noted for resonances of PRC carbons C-5 and C-6. This is similar to the behaviour of beta-blocker bases and salts reported by Zielinska-Pisklak et al. as investigated by solution-state NMR [39], which suggests the salt formation between PRO and CAP. The chemical shift of the carboxylic carbon may be used to assign the protonation or deprotonation state of the carboxyl group [40]. Carboxylic carbon (C-21) does not change significantly in PRC in comparison to CAP, but this is possible as the change in chemical shift associated with protonation/deprotonation is intrinsically limited by the fact that the principal elements of nuclear shielding tensors change in opposite directions and the total effect on chemical shift may not be observed [40]. However, a large downfield shift for alfa carbon (C-23) from 34.9 (CAP) to 40.0 ppm (PRC) was observed in the solid state. Similar changes were reported for propranolol laurate investigated by ^13^C solution-state NMR [38]; however, our solution-state studies did not reveal such a large difference, i.e., 34.12 and 34.87 ppm for CAP and PRC, respectively. Furthermore, the solution-state proton spectrum of PRC did not reveal signals arising from the NH group (*δ*_H_ = 1.55 ppm in the PRO spectrum) and COOH group (*δ*_H_ = 11.95 ppm in the PRO spectrum).

### 3.2. Solvent-Free Formation of PRC and Other Solid-State Forms of PRO-CAP

#### 3.2.1. Impact of Temperature and Mechanism of PRC Formation

**Figure 5:**
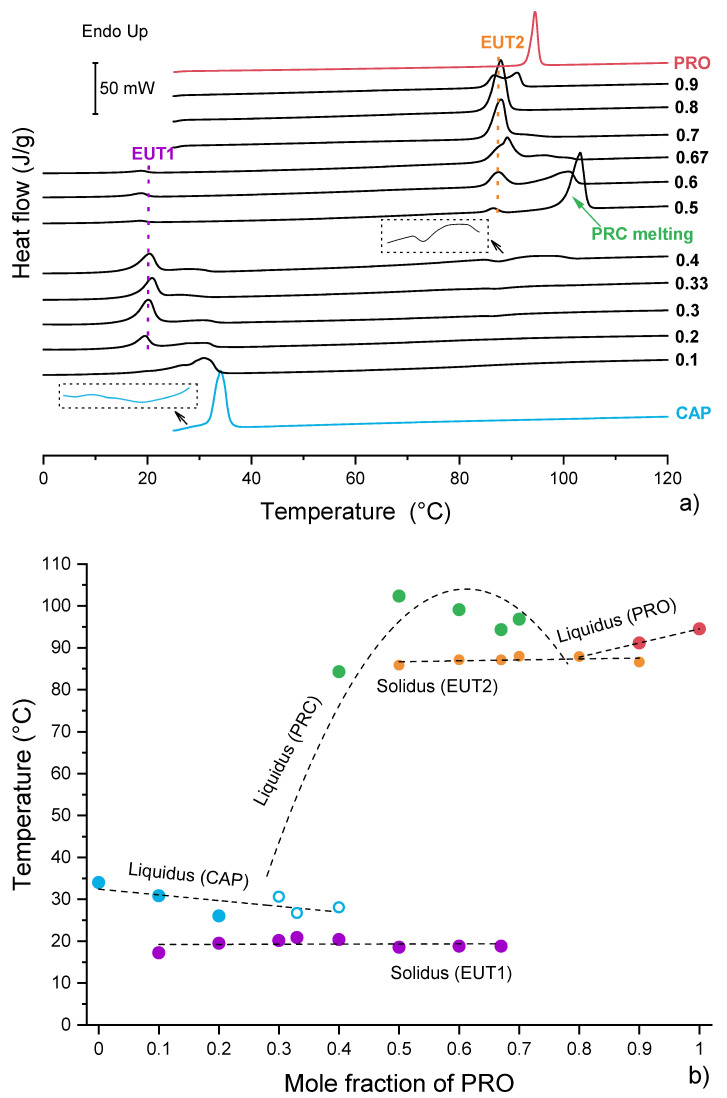
Thermal analysis of binary physical mixtures of PRO and CAP: (**a**) DSC traces of PRO−CAP physical mixture for first heating cycle. The labels represent mol fraction of PRO. (**b**) Temperature/composition phase diagram for the PRO−CAP system. Open symbols indicate uncertainty in identifying the phase. The broken lines are used as a guide for the eye.

Having successfully isolated and characterised the propranolol caprate salt, investigations were then directed to establish solvent-free synthesis of this multicomponent system. The method employed by Stott et al. involved dissolving PRO and CAP at different weight ratios in chloroform [13]. The solvent was then removed under vacuum, and samples were left at −20 °C until analysed. In contrast to Stott et al., we concentrated on a solvent-free approach [24,41]. The melting temperatures of pure PRO and CAP were observed at 91.8 °C and 31.9 °C, respectively. Upon a closer inspection of DSC thermograms, it was found that the peak assigned to CAP melting had an irregular baseline preceding the melting onset of CAP (Figure 5a), which may be assigned to a pre-melting phenomenon of CAP [42,43]. CAP as well as other long chain carboxylic acids may undergo a structural change on a thermal treatment, during which they lose crystalline structure and enter a liquid-like state.

Overall, the DSC traces of binary mixtures of PRO and CAP prepared without the use of solvent (Figure 5a) matched well those presented by Stott et al. [13]. An endotherm at 100 °C of a new phase (PRC) was observed at an equimolar composition. PRC has shown the ability to form eutectic phases with either PRO or CAP [13]. The DSC thermograms for the compositions below 0.5 mol fraction of PRO comprised an endotherm at a constant temperature of around 20 °C and peaks that could be assigned to either melting of PRC or excess of CAP. The endotherm at 20 °C was of the eutectic (EUT1) formed between PRC and CAP (Figure S7), as it has a lower melting point than either the components and it appears at a constant temperature. TGA analysis confirmed no weight loss in this region; thus, this peak was not associated with water loss (Figure S8). Based on the DSC data, the EUT1 composition is most likely to be around 0.33 mol of PRO, as at this composition a sharp endotherm is produced with the highest enthalpy of 38.4 J/g. The compositions above the equimolar (containing more than 0.5 mol fraction of PRO) showed an endotherm at a constant temperature of around 87 °C, which can be attributed to EUT2, formed between PRC and PRO (Figure S7). Similarly to the compositions below the equimolar, the melting points of the major components decrease as the eutectic composition is approached. The EUT2 composition is most likely to be around 0.67 mol fraction of PRO, as at this composition a sharp endotherm is produced with the highest enthalpy of 65.9 J/g. A closer inspection of the DSC traces also revealed a thermal event in the temperature range 75–100 °C for the sample containing 0.4 mol fraction of PRO. Further experiments by optical microscopy and PXRD revealed that this peak was of melting of residual PRO (Figure S9).

The major difference between our data (Figure 5) and those published by Stott and co-workers that can be observed is the DSC trace for the equimolar composition. For this sample, only one endotherm was seen by DSC [13]. However, in our case, three thermal events were recorded, with the first two endotherms attributable to EUT1 and EUT2, respectively. The last endotherm corresponded to the melting of PRC. The discrepancy in thermal data is reflected in the phase diagrams (Figure 5b and Figure S10) and can be attributed to the difference in sample preparation. Stott et al. [13] used a solvent for sample preparation, which allowed for a complete acid/base reaction, whereas our samples were prepared by mechanical mixing. Nevertheless, it can be concluded that the salt formation occurs in solution as well as in the solid state, as the presence of both eutectic phases in the thermogram of the equimolar composition signifies that PRC formed below 20 °C.

**Figure 6:**
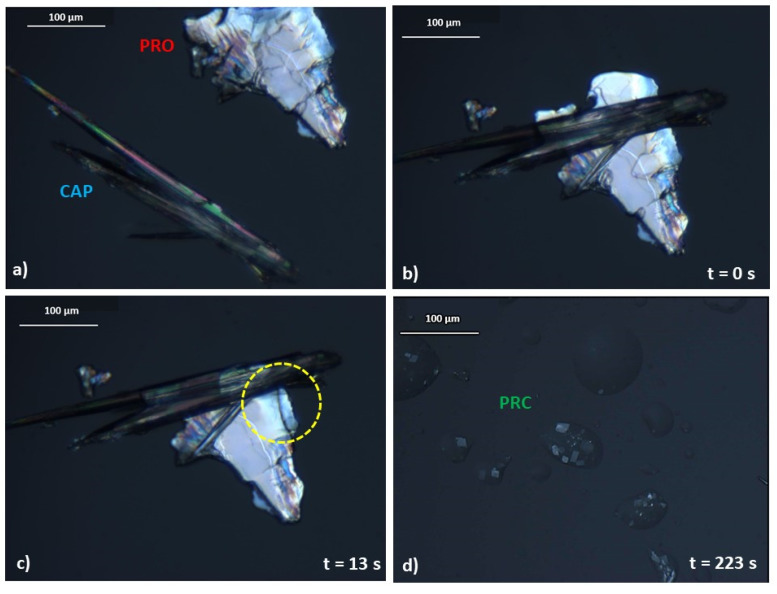
Images of polarised light microscopy experiment. The experiment was conducted at ambient temperature. (**a**) Crystal morphology of pure PRO and CAP. (**b**) PRO and CAP are brought into physical contact. (**c**) A liquid intermediate is formed after 13 s of bringing PRO and CAP into contact. The formation of a liquid intermediate is highlighted by a yellow circle. (**d**) After nearly 4 min, a new phase is formed.

Furthermore, an interesting observation was made during the preparation of the equimolar sample for DSC analysis, as it was observed that the sample felt and looked “wet”, as if a liquid had formed during mixing. A similar behaviour has previously been described by Chadwick et al. [16]. This group reported on mechanochemical preparation of a benzophenone–diphenylamine cocrystal. When crystals of benzophenone and diphenylamine were brought together at ambient conditions, a liquid phase formation was seen under the microscope, followed by growth of the new cocrystal from the liquid phase. Chadwick and co-workers proposed that the liquid phase was a submerged eutectic, which facilitated the formation of a cocrystal [16]. A similar phenomenon was seen but with lidocaine ionic liquids, and not a cocrystal, based on dicarboxylic acids [24]. Therefore, the hypothesis of a submerged eutectic being a precursor of PRC was tested using optical microscopy. When a crystal of PRO was brought into contact with a crystal of CAP at ambient temperature (Figure 6a), they began reacting immediately (Figure 6b) and a liquid phase was observed, marked by the yellow circle in Figure 6c. The growth of the salt crystals from the liquid phase was subsequently observed (Figure 6d). The crystal morphology of PRC differed significantly from the crystal shapes of pure PRO and CAP, and when the sample was heated, the newly formed phase began to melt at around 99 °C, which agreed with the DSC findings (Figure 5).

Therefore, it can be concluded that the reaction between CAP and PRO occurring in the solid state is facilitated by the formation of a metastable submerged eutectic, in addition to two other eutectic phases, EUT1 and EUT2, at higher temperatures identified by DSC (Figure 5a). This is also why no exotherm, leading to the salt formation, was recorded by DSC, as described for dipropranolol sebacate [25]. The temperature at which the eutectic between PRO and CAP would melt can be theoretically predicted using the Schroeder van Laar equation [44]. Using this equation, it was found that the calculated eutectic melting temperature would be around 30.5 °C, at 0.055 mol of PRO, (Figure S11), consistent with the low melting points of PRO and CAP. However, this approach does not take into account the possibility of component interaction, and the prediction of the eutectic temperature and composition may not be accurate. This is the case here, as the formation of a liquid intermediate strongly suggests that any eutectic phase between PRO and CAP forms below 30.5 °C.

#### 3.2.2. Mechanosynthesis of Propranolol Caprate Salt

In view of the above mechanism of PRO and CAP interaction via the submerged eutectic, the prospect of mechanosynthesis of PRC appeared quite attractive, as this method is solvent-free. During DSC experiments, it was observed that there were significant differences in thermograms for samples with the same composition, prepared and stored at 4 °C, but analysed at different timepoints after preparation. Figure S12 presents an example of this behaviour. When the system comprising 0.2 mol fraction of PRO was analysed immediately upon preparation, only melting of the eutectic and CAP was observed, whereas for the sample stored at 4 °C for 6 days, the melting endotherm of CAP was no longer seen and a new endotherm appeared, which may be attributed to melting of PRC. This confirms that the salt formation occurs even at 4 °C, following the co-grinding of reactants.

**Figure 7:**
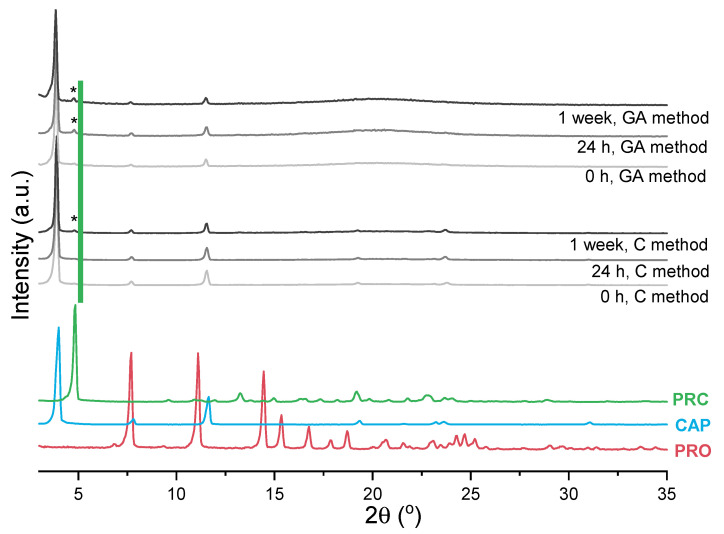
PXRD patterns of PRO, CAP (analysed as received), PRC (recrystallised from ethanol) and samples prepared by either co-grinding PRO and CAP in agate pestle and mortar at 0.2 mol of PRO at 4 °C (grinding-assisted (GA) method) or gentle mixing of PRO and CAP at 0.2 mol of PRO at 4 °C with a spatula at 4 °C (contact (C) method). Stars (*) indicate the characteristic peak of PRC.

The impact of sample preparation was also investigated and PRC formation monitored by PXRD to eliminate any unwanted transformations that might have been caused by the DSC treatment. The first approach, the contact method, involved gently mixing reactants, whereas the other involved co-grinding the reactants, similarly to the sample preparation for the DSC experiments. The gentle mixing method avoids input of thermal energy and loosening the molecules at the reaction site, as opposed to co-grinding [45]. Similarly to the DSC data presented in Figure S12, immediately upon mixing or co-grinding PRO and CAP at 0.2 mol fraction of PRO, salt formation was not observed. For the grinding-assisted method, the salt was detected 24 h after sample preparation, whereas for the contact method, it was detected after 1 week (Figure 7). To follow the PRC formation, the ratios of intensity of the peak at 4.8° 2theta (assigned to PRC) to the intensity of the peak at 3.9° 2theta (assigned to CAP) were calculated. It was found that after 1 week for the contact method, the ratio was 0.04, whereas for the grinding-assisted method, the ratio was 0.09. Additionally, the diffractograms of the samples prepared by grinding had distinct “halos”, indicating partial disordering/liquefaction of the components due to the use of mechanical force.

In conclusion, the formation of PRC via the submerged eutectic phase makes this salt an attractive multicomponent system that can be produced by mechanochemistry, solvent-free, and using a low energy input method, even by storing the equimolar PRO and CAP mixture at room temperature.

### 3.3. Characterisation of Supercooled PRO-CAP Systems

**Figure 8:**
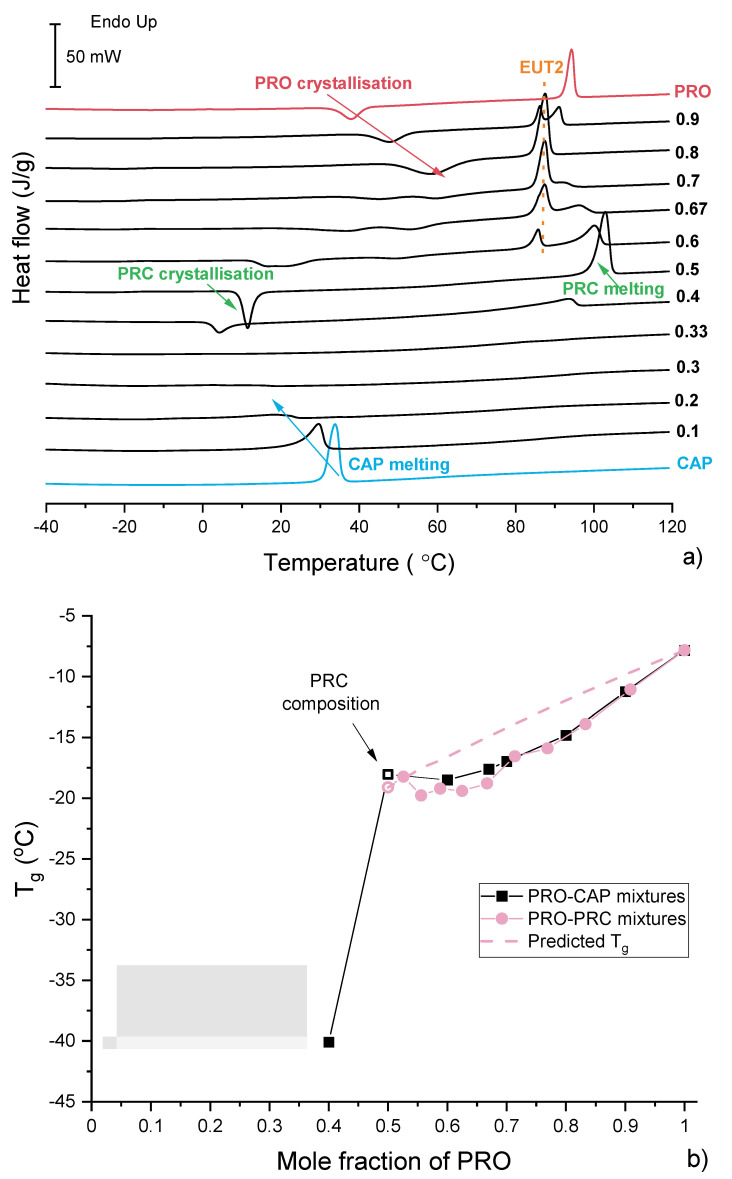
(**a**) DSC traces of PRO-CAP physical mixtures obtained in the second heating cycle. The labels represent mol fraction of PRO. (**b**) Glass transition temperature (T_g_) vs. composition relationship for supercooled PRO-CAP (black) and PRO-PRC (pink) mixtures. The predicted T_g_, shown as a broken pink line, was calculated using the Fox equation [46]. The solid lines are used as a guide for the eye.

The glass-forming ability and magnitude of the intermolecular forces was studied by quench cooling and reheating (by DSC) physical mixtures of PRO. TGAs of representative samples are shown in Figure S8. In general, it was found that pure PRO becomes supercooled, and its glass transition temperature (T_g_) was −7.8 °C, the onset of crystallisation was 33.6 °C and the crystallised sample melted at 92.3 °C (Figure 8a,b). CAP crystallised on cooling; therefore, its T_g_ could not be determined. The equimolar sample represents the salt composition, and it was found that this sample forms a glass with a T_g_ of −18.0 °C, indicating mixing of PRO and CAP at the molecular level following melting of both components. The compositions containing excess CAP also crystallised on cooling, and hence no glass-transition (T_g_) was observed for these samples (Figure 8a,b). Weak in magnitude thermal events were seen for the samples at 0.3 and 0.33 mol fraction of PRO. No EUT1 was observed on DSC thermograms of the supercooled samples, suggesting their good thermal stability.

The samples containing more than 0.4 mol fraction of PRO became supercooled, and the system at 0.4 mol fraction of PRO had the lowest T_g_. The plot of T_g_ versus composition is shown in Figure 8b. A significant T_g_ increase was noted going from 0.4 to 0.5 mol fraction of PRO, most likely due to strong intermolecular forces present in the equimolar sample [25]. The T_g_ value then slightly increased until the T_g_ of pure PRO was reached. For the samples containing excess PRO, the melting of EUT2 was observed during the reheating. The melting of EUT2 was observed as PRC and PRO had crystallised below the EUT2 melting temperature and were able to form this eutectic.

While SCXRD confirmed the ionic nature of the salt in the crystalline phase and thermal analysis indirectly implied strong intermolecular interactions in the equimolar supercooled phase (Figure 8b), a detailed investigation into the ionic nature of the salt was carried out by broadband dielectric spectroscopy (BDS). The physical parameter that is the most commonly employed to describe the ionic nature of a system is dc-conductivity (σ_dc_), usually defined as the number of ions multiplied by their mobility. For systems composed solely of ions, σ_dc_ values of 10^−3^–10^−5^ S/cm are typically measured at room temperature and 10^−14^–10^−15^ S/cm in the vicinity of a liquid–glass transition. On the other hand, partially ionised compounds with efficient proton transport between acid and base show σ_dc_(T_g_) values higher than 10^−14^ S/cm. Thus, σ_dc_ is considered as a useful factor describing the charge transport mechanism in protic ionic liquids. 

**Figure 9:**
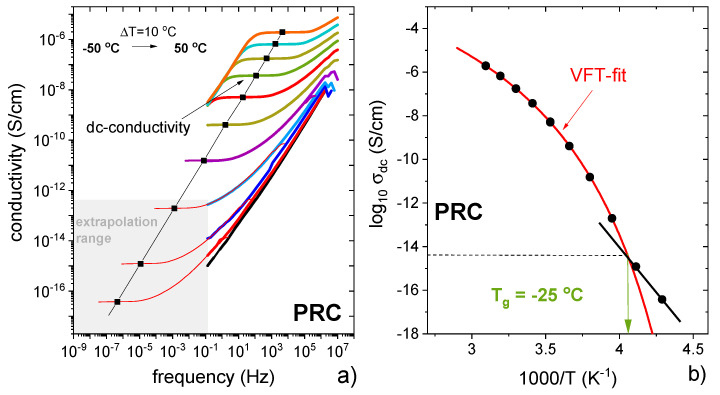
(**a**) Dielectric data of PRC presented in conductivity representation. (**b**) Temperature dependence of dc-conductivity of PRC.

Supercooled PRC was prepared by melting at 100 °C and cooled down to −50 °C, i.e., approx. 30 degrees below its calorimetric T_g_. According to the standard practice, the dielectric spectra are presented in the conductivity σ*(f) formalism [47,48]. As can be seen in Figure 9a, the real part of the complex conductivity function recorded in the frequency range 10^−1^–10^6^ Hz reveals a step-like behavior, with the plateau region corresponding to σ_dc_. It is evident that σ_dc_ decreased during the isobaric cooling, which is typical for ion-conducting materials. The temperature difference of 100 °C reduced σ_dc_ by around ten orders of magnitude. This is visualised in Figure 9a, where the temperature evolution of σ_dc_ is presented. It can be seen that log_10_σ_dc_ vs. T^−1^ shows a clear crossover from a super-Arrhenius to Arrhenius-like behavior, which indicates a transition of PRC from a supercooled liquid to the glassy state [49]. The liquid–glass transition temperature, T_g_, determined from this crossover, was −25 °C (Figure 9b), and it is broadly consistent with the value obtained from the thermal experiments (−18 °C). The difference can be explained by different heating rates applied in DSC and BDS scans (10 °C/min vs. 5 °C/min, respectively). Importantly, for PRC, σ_dc_(T_g_) is approximately equal to 10^−14^ S/cm, which indicates full coupling between charge transport and structural dynamics in this compound [48]. In other words, the ion transport is controlled only by translational motions of PRO^+^ cations and CAP^-^ anions. Additionally, the dielectric data indicate no proton transport contribution between PRO and CAP to overall dc-conductivity.

### 3.4. Analysis of Eutectic Compositions

**Figure 10:**
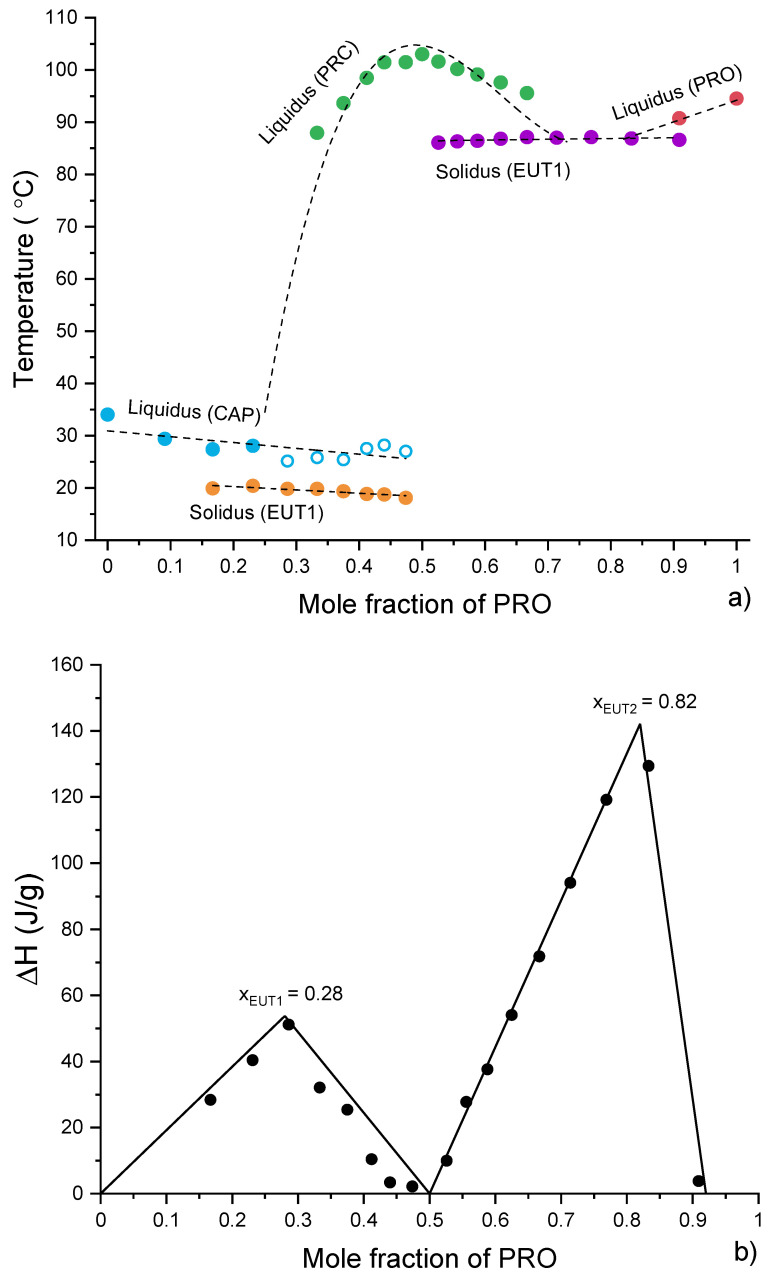
(**a**) Phase diagram constructed using thermal data obtained from the first heating of physical mixtures (CAP-PRC and PRO-PRC). Open symbols indicate uncertainty in identifying the phase. The lines are used as a guide for the eye. (**b**) Tammann plots constructed using thermal data obtained from the first heating of physical mixtures (CAP-PRC and PRO-PRC). The lines are best fits to the data points.

Thermal analysis of CAP-PRC and PRO-PRC physical mixtures was conducted to gain insight into the formation of the eutectic phases (Figure S13a,b). A phase diagram was constructed on the basis of full thermal analysis of CAP-PRC and PRO-PRC mixtures (Figure 10a). It confirms the formation of EUT1 between CAP and PRC and EUT2 between PRO and PRC. However, the position and/or identity of some of the peaks at high CAP content could not be resolved (Figure 10a). The Tammann plot (Figure 10b) showed that, based on the enthalpy of melting of the eutectic phases, EUT1 comprises 0.28 mol fraction of PRO, while EUT2 comprises 0.82 mol fraction of PRO.

Reheated quench-cooled CAP-PRC and PRO-PRC physical mixtures showed thermal features similar to those of the PRO-CAP systems with equivalent compositions (Figure S13c,d). The samples containing an excess of acid crystallised on cooling. Samples at 0.333 and 0.286 mol fraction of PRO had overlapping peaks of PRC recrystallisation and CAP melting. The samples containing 0.5 and higher PRO mol fractions were able to quench cool, and the plot of T_g_ values against the composition can be seen in Figure 8b. It is clear that the points do not follow the Fox prediction and are located below the theoretical line, constructed using T_g_s of PRO and PRC. Unfortunately, T_g_ values below the equimolar composition of PRO and CAP cannot be predicted as the T_g_ of pure CAP is unknown.

**Figure 11:**
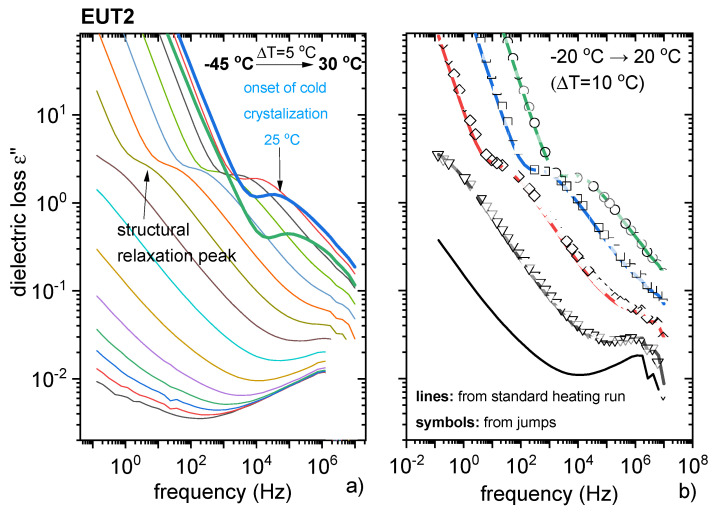
Dielectric studies of EUT2. (**a**) Data recorded on a heating scan. (**b**) Comparison between dielectric spectra of EUT2 measured applying two different procedures (description in text).

The ionic nature of the supercooled EUT1 composition could not be investigated by BDS, as this system is unable to supercool. Thus, BDS studies were only performed on supercooled EUT2, as this system is a mixture of a neutral compound (PRO) and PRC and can reveal either ionic or non-ionic character. In analogy to the experimental protocol applied for pure PRC, EUT2 was first melted at 87 °C and subsequently quenched to T = T_g_−30 °C. Interestingly, the dielectric results of EUT2 assume a pattern characteristic for van der Waals liquids rather than ionic systems [32]. The σ_dc_ contribution to the overall dynamics of EUT2 was low enough to detect its structural relaxation process. These processes can be easily distinguished when the dielectric data of EUT2 are presented in permittivity ε * (f) formalism [32]. In Figure 11, it can be seen that above the T_g_, the imaginary part of complex permittivity function ε”(f) takes the form of a peak (corresponding to the structural relaxation process) that is affected by a rapid increase in the ε(f) function on the low-frequency side (σ_dc_ contribution). The structural relaxation process (so called α-relaxation), related to the cooperative rearrangements of molecules, accelerates with heating. However, from Figure 11a it is evident that at 25 °C, the dielectric strength of this mode begins to decrease rapidly. This behaviour manifests the cold crystallisation of EUT2. It needs to be noted that during the BDS experiment, the cold crystallisation process occurred at a much lower temperature than observed in the DSC studies. The reason for this behaviour can be two-fold: different heating rates in DSC and DBS experiments or partial crystallization of sample during the quenching process. To verify which scenario is true, an additional BDS experiment was performed. The dielectric data of EUT2 were recorded at the same temperature conditions as before; however, the melting process was approached in between. As presented in Figure 11b, both procedures, i.e., standard heating from the glassy state as well as T_1_-T_m_-T_2_-T_m_-… jumps gave the same results. Consequently, the difference in crystallisation temperature is most likely due to the different heating rates applied in both experiments.

Since the maxima of α-relaxation peaks are not well pronounced in EUT2, the structural relaxation times were determined by fitting experimental data with the Cole–Davidson function. The obtained temperature variation of τ_α_ is depicted in Figure S14. As can be seen, logτ_α_(T^−1^) points of EUT2 reveal a non-linear behaviour, well parameterised by means of the empirical Vogel–Fulcher–Tammann (VFT) equation [32]. The extrapolation of the VFT function to 100 s was applied to determine the T_g_ of EUT2, which was determined to be −20 °C and is in relatively good agreement with DSC data. The results for EUT2, quite different from those obtained for PRC, clearly indicate that in EUT2 neutral PRO dominates the molecular dynamics, while the contribution of PRC is minimal. 

This rather non-ionic character of EUT2 might be of advantage in transdermal applications of PRO. Green and Hadgraft [50] as well as Stott et al. [13] postulated that the transport of cationic drugs (PRO) was enhanced by an ion-pair mechanism in the presence of fatty acids and that the said presence of the fatty acid increased lipophilicity of the system. Following a melting temperature-membrane transport (MTMT) model [51], based on the ideal solution theory to predict relative transdermal fluxes of compounds with different melting points, it can be hypothesised that EUT2, with a melting point of around 87 °C, as opposed to PRC melting at app. 100 °C, coupled with the unionised PRO molecules having greater permeation due to the hydrophobic similarity with the stratum corneum [52], might have better transdermal transport.

## 4. Conclusions

The crystal structure of the salt formed between PRO and CAP–propranolol caprate was solved, confirming that the salt stoichiometry is 1:1. This research generated insight into fundamental understanding of this salt mechanism, which is via a submerged eutectic, formed between PRO and CAP. The salt was prepared by classical solvent crystallisation; however, due to this unique mechanism of formation, PRC is a very good candidate for the mechanochemical route of manufacturing, making the synthesis more environmentally friendly. The salt formation was observed even at 4 °C, which is considerably below the melting point of the reactants. In addition to the submerged eutectic phase, two other, solid at room temperature, eutectics were confirmed, composed of PRC and either CAP or PRO, at 0.28 and 0.82 mol fraction of PRO, respectively. Broadband dielectric spectroscopy indicated that the supercooled PRC has ionic character, whereas the supercooled EUT2 had predominantly non-ionic properties despite comprising the salt.

## Data Availability

The data presented in this study are available on request from the corresponding author.

## Supplementary Materials

The following are available online at https://www.mdpi.com/article/10.3390/pharmaceutics13122125/s1, Table S1. Hydrogen bond lengths for PRC. Table S2. Solution-state NMR (DMSO-*d_6_*) ^1^H chemical shifts for CAP. Table S3. Solution-state NMR (DMSO-*d_6_*) ^1^H chemical shifts for PRO. Table S4. Solution-state NMR (DMSO-*d_6_*) ^1^H chemical shifts for PRC. Table S5. Solution and solid-state NMR ^13^C chemical shifts for PRO, PRC and CAP. Figure S1. Individual disordered capric acid moieties in PRC unit with O20D–C31E occupied 27%. Displacement shown at 50% probability. Hydrogen bonding shown as red dotted lines. Figure S2. Le Bail refinement for the PXRD pattern of PRC calculated from SCXRD (red) and that determined by PXRD (black). Figure S3. Carbon-13 (100.61 MHz) spectra (spectral editing) of PRO (A) standard CPMAS (B) with polarisation inversion time 60 μs. Recorded with a spinning rate of 20 kHz. Polarisation inversion time was set in order to obtain quaternary C and CH3 positive, CH2 negative and CH ~null. Figure S4. Carbon-13 (100.61 MHz) spectra (spectral editing) of PRC (A) standard CPMAS and with polarisation inversion time (B) 60 μs (C) 80 μs (D) 100 μs. Recorded with a spinning rate of 20 kHz. Generally, polarisation inversion time was set in order to obtain quaternary C and CH3 positive, CH2 negative and CH ~null. However, with polarisation inversion 60 μs, CH2 arising from caprate are positive; with 80 μs, only quaternary C and CH3 are positive and CH/CH2 negative; with 100 μs, only CH3 from caprate and all quaternary C are positive. Figure S5. (A) Low-frequency and (B) high-frequency region of ^1^H–^13^C HETCOR spectrum of PRO with contact time of 0.1 ms showing directly bonded C–H links only. Figure S6. (A) Low-frequency and (B) high-frequency region of ^1^H–^13^C HETCOR spectrum of PRC with contact time of 0.1 ms showing directly bonded C–H links only. Figure S7. PXRD pattern of PRO, CAP, PRC, EUT1 and EUT2. The PXRD pattern of EUT1 consists of peaks corresponding to PRC and CAP only, while the PXRD pattern of EUT2 consists of peaks corresponding to PRC and PRO only. Figure S8. Thermogravimetric analysis (TGA) data for PRO, CAP, PRC, EUT1 and EUT2. Figure S9. PXRD pattern of PRO, CAP, PRC and the physical mixture of PRO and CAP at 0.4 mol fraction of PRO. The PXRD pattern of the sample comprising 0.4 mol fraction of PRO consists of peaks of the starting materials or salt only. Figure S10. Temperature/composition phase diagram for the PRO-CAP system based on data published by Stott et al. [1], obtained from Elsevier with permission. Figure S11. Theoretical prediction of temperature and composition of the eutectic formed between PRO and CAP. The theoretical liquidus curves were calculated using the Schroeder van Laar equation. Using this equation, it was found that the calculated eutectic melting temperature is around 30.5 °C and this eutectic phase would comprise 0.055 mol of PRO. Figure S12. DSC thermograms of CAP (analysed as received), PRC (crystallised from ethanol) and samples prepared by co-grinding PRO and CAP in an agate mortar at 0.2 mol fraction of PRO at 4 °C. Figure S13. (a) DSC traces of the CAP-PRC physical mixtures from the first heating cycle; (b) DSC traces of the PRO-PRC physical mixtures from the first heating cycle; (c) DSC 2nd heating cycle thermograms of CAP-PRC systems and (d) DSC 2nd heating cycle thermograms of PRO-PRC systems. The labels represent the mol fraction of PRO. Figure S14. Relaxation map of EUT2. The solid line denotes VFT fit of experimental data.
