# Supplementary material for: Submerged Eutectic-Assisted, Solvent-Free Mechanochemical Formation of a Propranolol Salt and Its Other Multicomponent Solids
Source: Pharmaceutics. 2021 Dec 9;13(12):2125. doi: 10.3390/pharmaceutics13122125 (PMC8703429; doi:10.3390/pharmaceutics13122125)
Supplement: Supplementary file 1 [file pharmaceutics-13-02125-s001.zip › pharmaceutics-1475260-supplementary.pdf]

pharmaceutics

Supplementary Materials: Submerged Eutectic-Assisted, Solvent-Free Mechanochemical Formation of a Propranolol Salt and Its Other Multicomponent Solids

Klaudia Bialek, Zaneta Wojnarowska, Marcin Skotnicki, Brendan Twamley, Marian Paluch, Lidia Tajber

Table S1. Hydrogen bonds lengths for PRC.

D-H...A	d(D-H)	d(H...A)	d(D...A)	<(DHA)
O(7A)-H(7A)...O(20A)	0.84	1.86	2.6960(14)	173
N(4A)-H(4AA)...O(20A)#1	0.91	1.87	2.7816(14)	175
N(4A)-H(4AB)...O(22A)	0.91	1.82	2.7216(15)	169
O(7B)-H(7B)...O(22B)	0.84	1.85	2.6886(14)	176
N(4B)-H(4BA)...O(20B)	0.91	1.87	2.7626(15)	168
N(4B)-H(4BB)...O(20C)	0.91	1.86	2.7736(14)	176
O(7C)-H(7C)...O(20C)	0.84	1.84	2.6759(14)	175
N(4C)-H(4CA)...O(22B)	0.91	1.83	2.7403(14)	176
N(4C)-H(4CB)...O(22C)	0.91	1.84	2.7340(15)	168
O(7D)-H(7D)...O(22D)	0.84	1.84	2.6782(14)	177
N(4D)-H(4DA)...O(20D)	0.91	1.86	2.7588(16)	168
N(4D)-H(4DB)...O(22D)#2	0.91	1.84	2.7504(14)	176

Symmetry transformations used to generate equivalent atoms:

#1 -x+2,-y+1,-z+1 #2 -x,-y+1,-z

Table S2. Solution-state NMR (DMSO-d_6) ^{1}H chemical shifts for CAP.

<table>
<tr><th>Atom number</th><th>δ_H / ppm</th></tr>
<tr><td>31; –CH$_2$–CH$_3$</td><td>0.86 (t, 3H)</td></tr>
<tr><td>24; –CH$_2$–CH$_2$–COOH</td><td>1.49 (p, 2H)</td></tr>
<tr><td>30; –CH$_2$–CH$_3$</td><td rowspan="5">1.25 (m, 12H)*</td></tr>
<tr><td>25; –CH$_2$–(CH$_2$)$_2$–COOH</td></tr>
<tr><td>28, 26;–CH$_2$–CH$_2$–CH$_2$–(CH$_2$)$_3$–COOH</td></tr>
<tr><td>27; –CH$_2$–CH$_2$–CH$_2$–(CH$_2$)$_3$–COOH</td></tr>
<tr><td>29; –CH$_2$–CH$_2$–CH$_3$</td></tr>
<tr><td>23; –CH$_2$–COOH</td><td>2.19 (t, 2H)</td></tr>
<tr><td>21; –COOH</td><td>11.95 (br s, 1H)</td></tr>
</table>

*signals overlapped

Table S3. Solution-state NMR (DMSO-d_6) ^{1}H chemical shifts for PRO.

Atom number	δ_H / ppm
1, 2; –CH$_2$–NH–CH–(**CH$_3$**)$_2$	1.00, 0.99 (2d, 6H)
5; –**CH$_2$**–NH–CH–(CH$_3$)$_2$	2.69, 2.80 (2dd, 2H)
3; –CH$_2$–NH–C**H**–(CH$_3$)$_2$	2.74 (sep, 1H)
6; –O–CH$_2$–C**H**–OH	4.01 (m, 2H)
8; –O–C**H$_2$**–CH–OH	4.07, 4.13 (2dd, 2H)
11; Ar**H**	6.96 (d, 1H)
13; Ar**H**	7.45 (d, 1H)
18; Ar**H**	8.23 (dd, 1H)
17; Ar**H**	7.51 (m, 1H)*
12; Ar**H**	7.41 (t, 1H)
16; Ar**H**	7.51 (m, 1H)*
15; Ar**H**	7.87 (d, 1)
N(4)**H**	1.55 (br s, 1)
O(7)**H**	5.09 (br d, 1)

*signals overlapped

Table S4. Solution-state NMR (DMSO-d_6) ^{1}H chemical shifts for PRC.

Atom number	δ_H / ppm
1, 2; –CH$_2$–NH–CH–(C**H**$_3$)$_2$	1.04 (d, 6H)
5; –C**H**$_2$–NH–CH–(CH$_3$)$_2$	2.74, 2.85 (2dd, 2H)*
3; –CH$_2$–NH–C**H**–(CH$_3$)$_2$	2.83 (sep, 1H)
6; –O–CH$_2$–C**H**–OH	4.07 (m, 2H)*
8; –O–C**H**$_2$–CH–OH	4.07, 4.13 (2dd, 2H)*
11; Ar**H**	6.96 (d, 1H)
13; Ar**H**	7.46 (d, 1H)
18; Ar**H**	8.23 (dd, 1H)
17; Ar**H**	7.51 (m, 1H)[a]
12; Ar**H**	7.41 (t, 1H)
16; Ar**H**	7.51 (m, 1H)
15; Ar**H**	7.87 (d, 1)
N(4)**H**	Not observed
O(7)**H**	Not observed
31; –CH$_2$–C**H**$_3$	0.84 (t, 3H)
24; –C**H**$_2$–CH$_2$–COOH	1.48 (p, 2H)
30; –C**H**$_2$–CH$_3$	1.24 (m, 12H)*
25; –C**H**$_2$–(CH$_2$)$_2$–COOH	
28, 26; –C**H**$_2$–CH$_2$–C**H**$_2$–(CH$_2$)$_3$–COOH	
27; –CH$_2$–C**H**$_2$–CH$_2$–(CH$_2$)$_3$–COOH	
29; –C**H**$_2$–CH$_2$–CH$_3$	
23; –C**H**$_2$–COOH	2.14 (t, 2H)
21; –COO**H**	Not observed

*signals overlapped

Table S5. Solution and solid-state NMR ^{13}C chemical shifts for PRO, PRC and CAP.

Carbon number	PRO		PRC		CAP	
	^{13}C solution-state NMR δ_C / ppm	^{13}C CPMAS δ_C / ppm	^{13}C solution-state NMR δ_C / ppm	^{13}C CPMAS δ_C / ppm	^{13}C solution-state NMR δ_C / ppm	^{13}C CPMAS δ_C / ppm
1, 2; –CH$_2$–NH–CH–(**C**H$_3$)$_2$	22.95	21.6, 24.7	22.64, 22.74	16.2, 19.6	N/A	
3; –CH$_2$–NH–**C**H–(CH$_3$)$_2$	48.17	48.9	48.86	49.8*		
5; –**C**H$_2$–NH–CH–(CH$_3$)$_2$	50.05	51.8	50.07			
6; –O–CH$_2$–**C**H–OH	68.48	69.2	68.41	67.1		
8; –O–**C**H$_2$–CH–OH	70.97	70.2	71.34	68.9		
11; ArH	105.12	103.0	105.64	106.7		
13; ArH	119.77	Maxima at* 119.0, 120.6, 124.2, 125.8	120.34	Maxima at* 120.3, 121.7, 125.2, 127.7		
18; ArH	121.72		122.23			
17; ArH	125.10		125.62			
12; ArH	126.21		126.70			
16; ArH	126.37		126.89			
15; ArH	127.35		127.86			
19; Ar	124.99	124.6	125.48	125.3		
14; Ar	133.99	133.6	134.50	134.5		
10; ArO	154.16	152.4	154.61	153.5		
31; –CH$_2$–**C**H$_3$	N/A		14.42	14.7	14.41	15.0
30; –**C**H$_2$–CH$_3$			22.56	23.8,	22.56	25.0
24; –**C**H$_2$–CH$_2$–COOH			25.28	Maxima at* 26.8, 30.2, 31.0, 31.8 32.9	24.96	Maxima at* 31.6, 32.9, 33.4
25; –**C**H$_2$—(CH$_2$)$_2$–COOH			29.14		29.01	
28, 26; –**C**H$_2$–CH$_2$–**C**H$_2$—(CH$_2$)$_3$–COOH			29.15; 29.28		29.12; 29.21	
27; –CH$_2$—**C**H$_2$–CH$_2$—(CH$_2$)$_3$–COOH			29.38		29.34	
29; –**C**H$_2$–CH$_2$–CH$_3$			31.75		31.74	
23; –**C**H$_2$–COOH			34.87	40.0	34.12	34.9
21; –**C**OOH			175.63	182.3	174.95	181.9

*signals overlapped

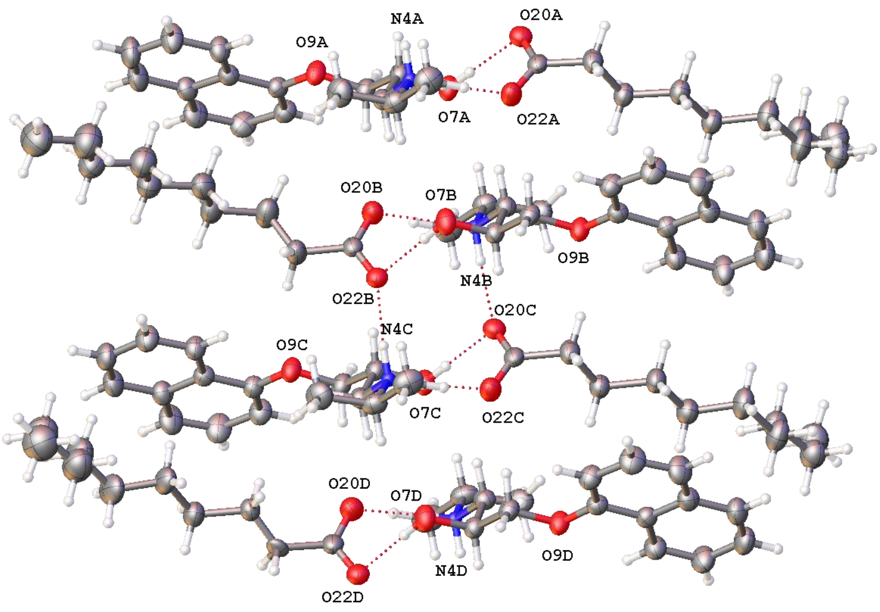

Figure S1. Individual disordered capric acid moieties in PRC unit with O20D – C31E occupied 27%. Displacement shown at 50% probability. Hydrogen bonding shown as red dotted lines.

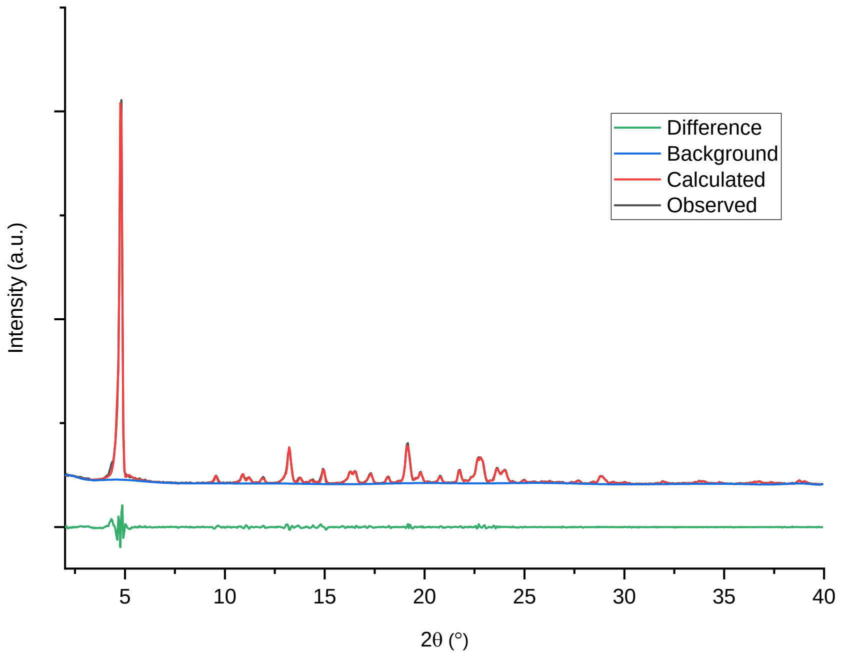

Figure S2. Le Bail refinement for the PXRD pattern of PRC calculated from SCXRD (red) and that determined by PXRD (black).

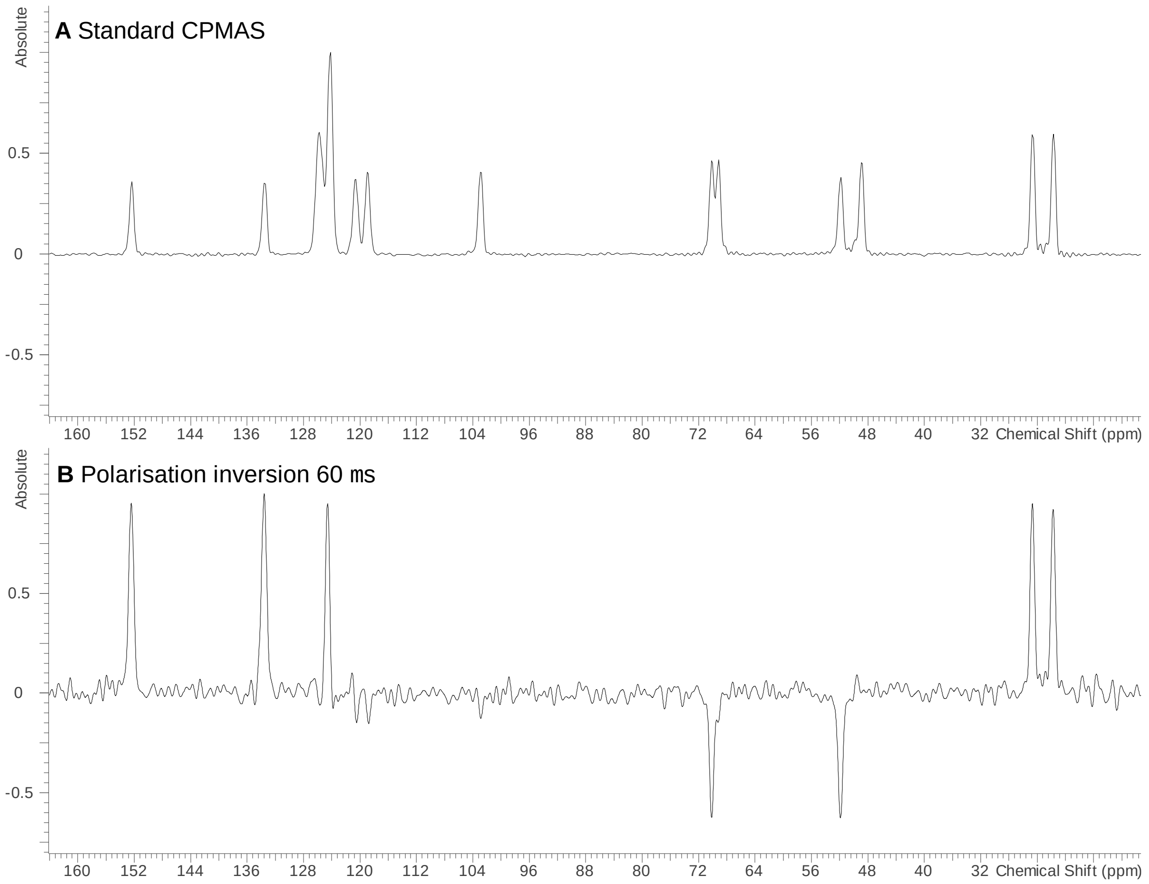

Figure S3. Carbon-13 (100.61 MHz) spectra (spectral editing) of PRO (A) standard CPMAS (B) with polarisation inversion time 60 μs. Recorded with a spinning rate of 20 kHz. Polarisation inversion time was set in order to obtain quaternary C and CH_3 positive, CH_2 negative and CH ~null.

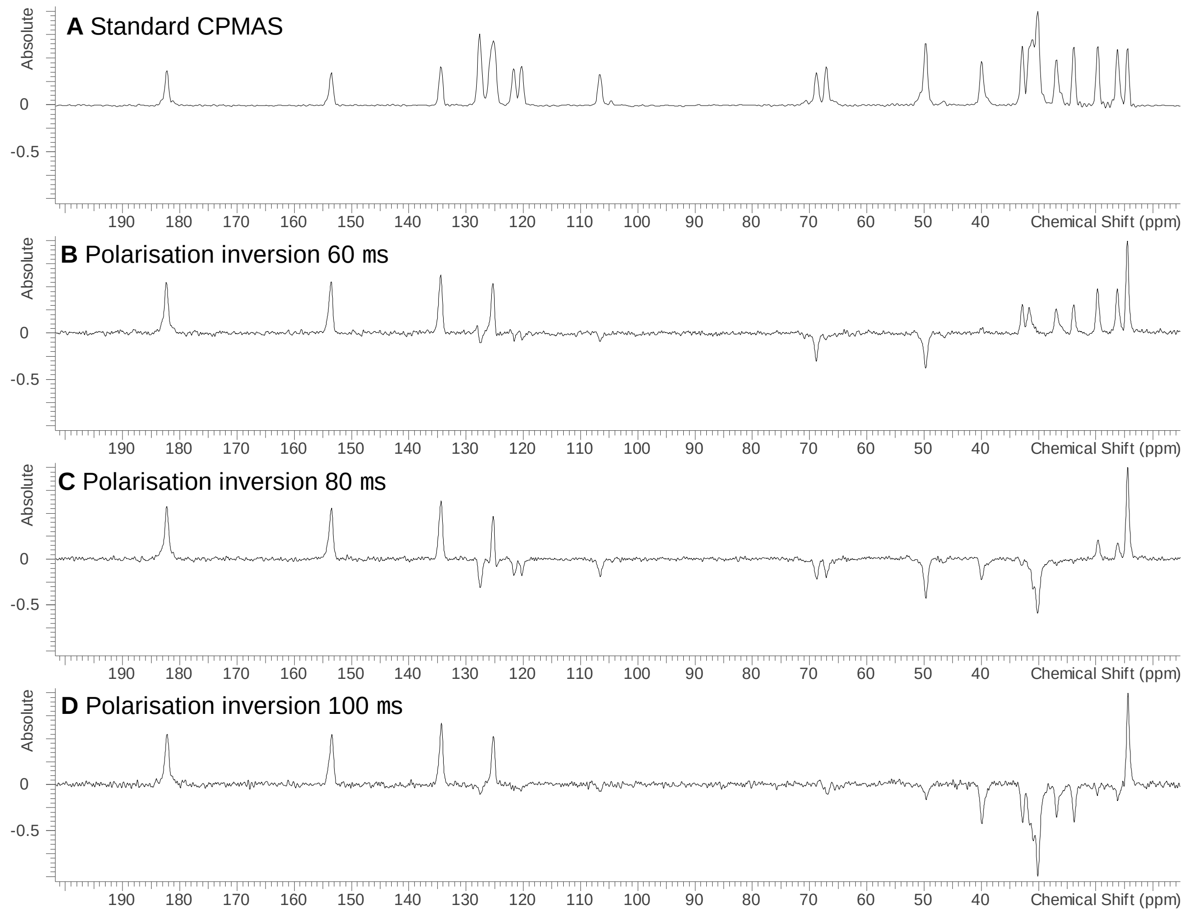

Figure S4. Carbon-13 (100.61 MHz) spectra (spectral editing) of PRC (A) standard CPMAS and with polarisation inversion time (B) 60 μs (C) 80 μs (D) 100 μs. Recorded with a spinning rate of 20 kHz. Generally, polarisation inversion time was tried to set in order to obtain quaternary C and CH_3 positive, CH_2 negative and CH ~null. However, with polarisation inversion 60 μs CH_2 arising from caprate are positive, with 80 μs only quaternary C and CH_3 are positive and CH/CH_2 negative, with 100 μs only CH_3 from caprate and all quaternary C are positive.

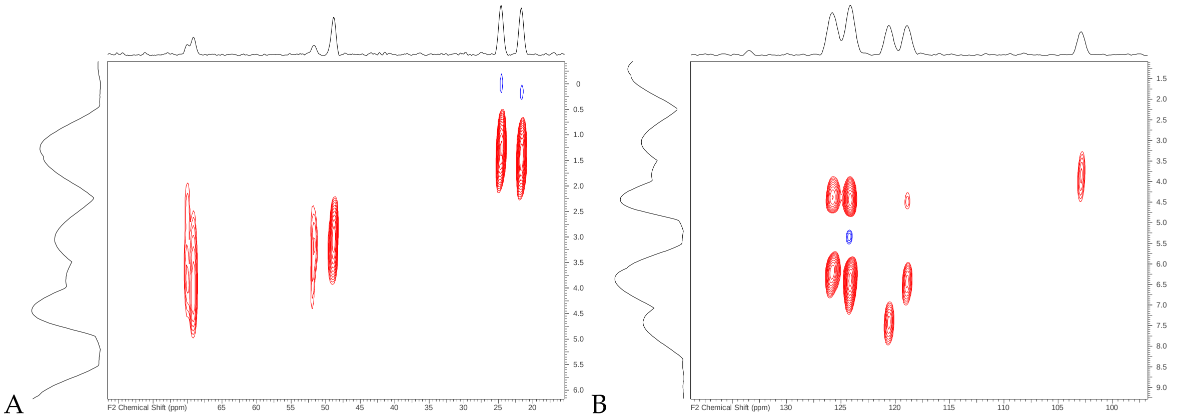

Figure S5. (A) Low-frequency and (B) high-frequency region of ^{1}H–^{13}C HETCOR spectrum of PRO with contact time of 0.1 ms showing directly bonded C-H links only.

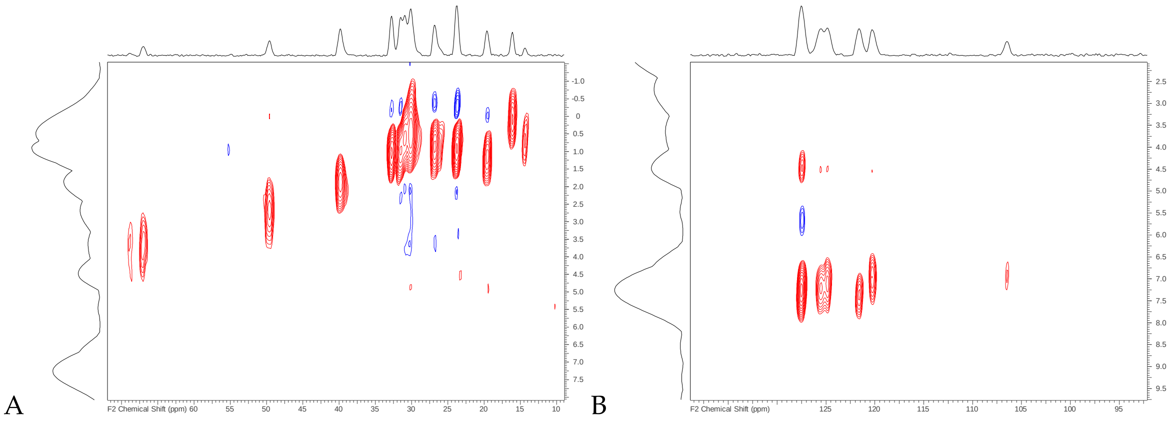

Figure S6. (A) Low-frequency and (B) high-frequency region of ^{1}H–^{13}C HETCOR spectrum of PRC with contact time of 0.1 ms showing directly bonded C-H links only.

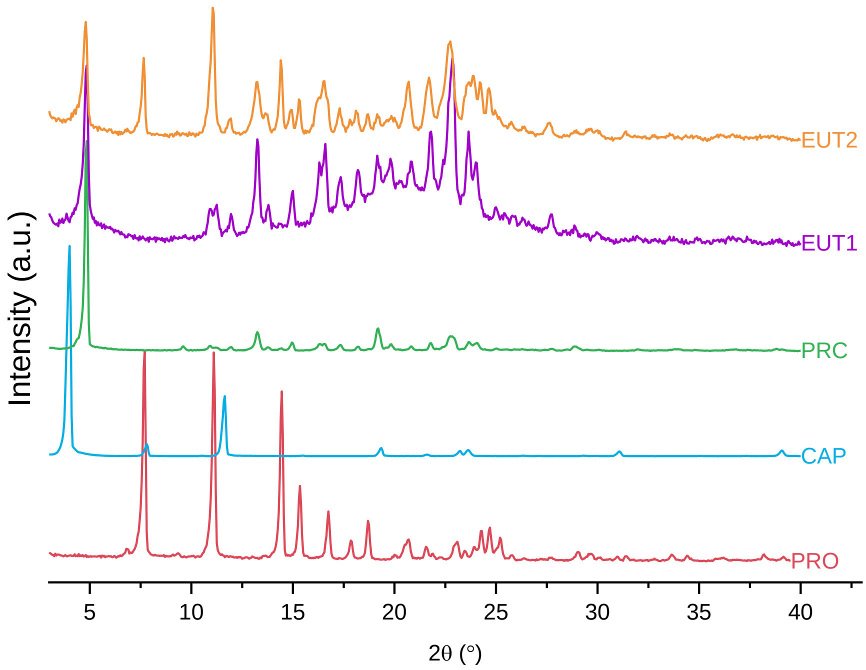

Figure S7. PXRD pattern of PRO, CAP, PRC, EUT1 and EUT2. The PXRD pattern of EUT1 consists of peaks corresponding to PRC and CAP only, while the PXRD pattern of EUT2 consists of peaks corresponding to PRC and PRO only.

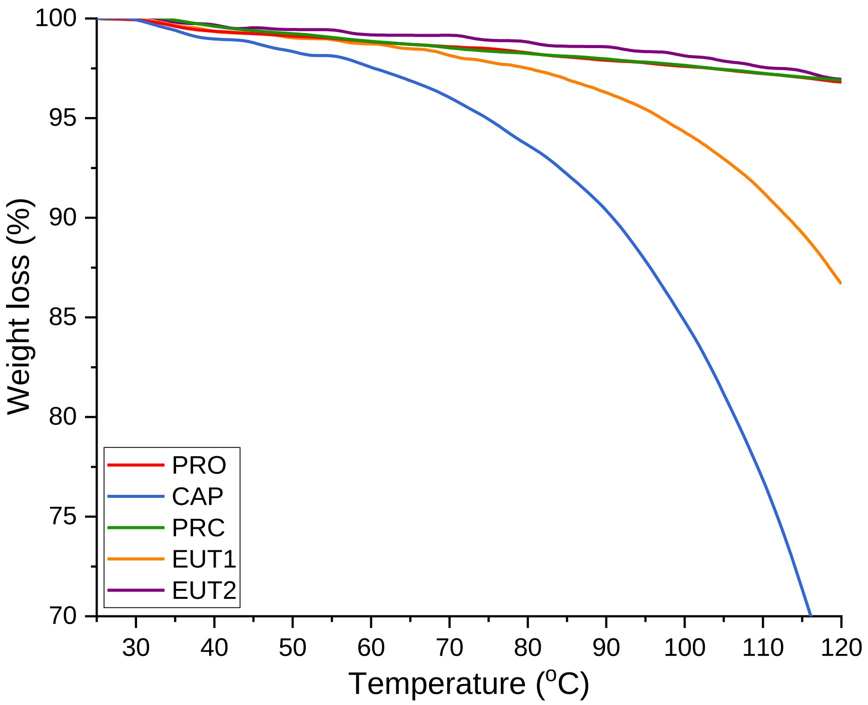

Figure S8. Thermogravimetric analysis (TGA) data for PRO, CAP, PRC, EUT1 and EUT2.

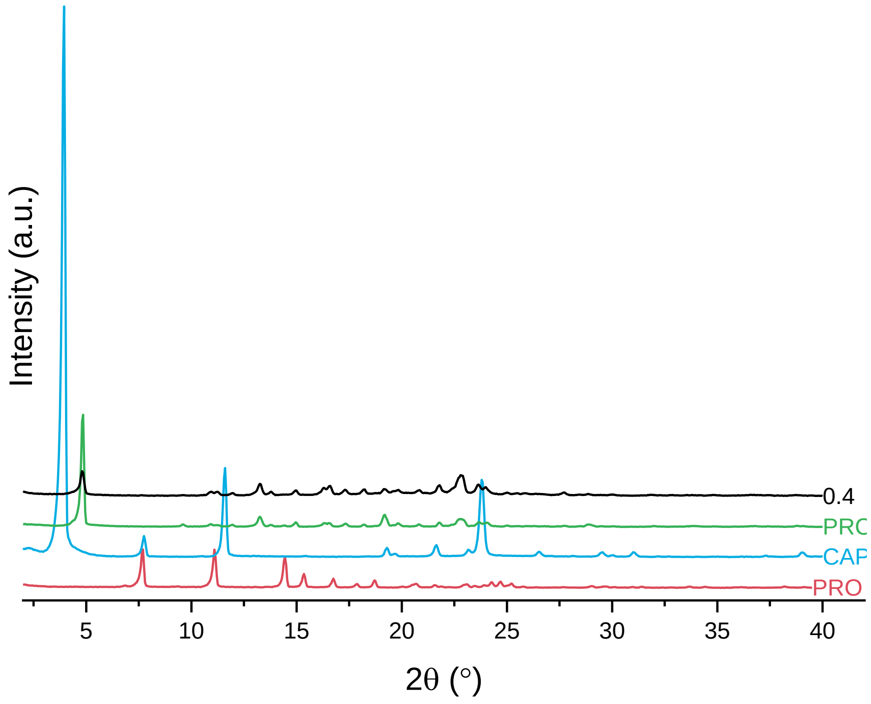

Figure S9. PXRD pattern of PRO, CAP, PRC and the physical mixture of PRO and CAP at 0.4 mol fraction of PRO. The PXRD pattern of the sample comprising 0.4 mol fraction of PRO consists of peaks of the starting materials or salt only.

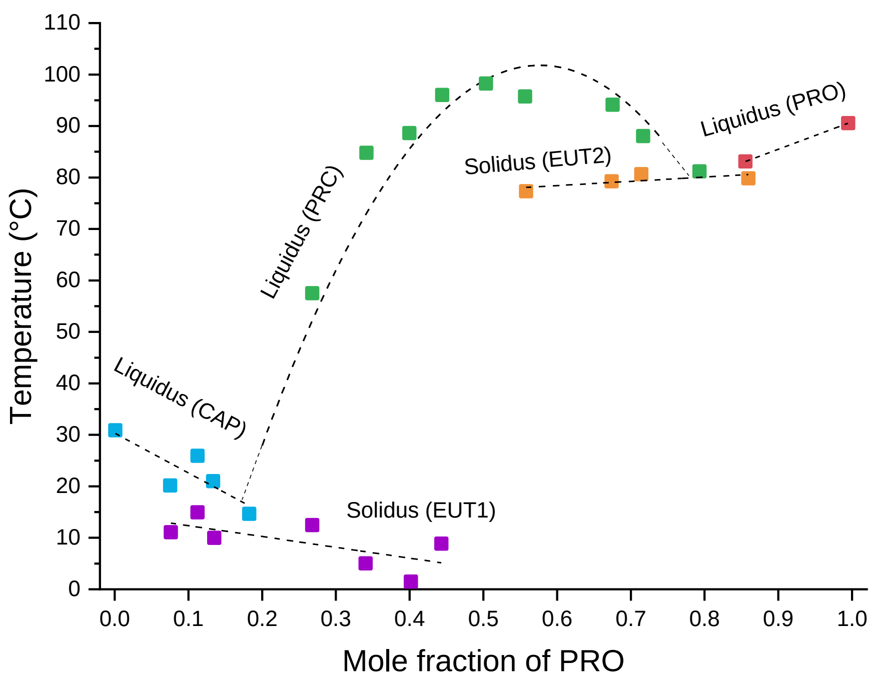

Figure S10. Temperature/composition phase diagram for the PRO-CAP system based on data published by Stott et al. [1], obtained from Elsevier with permission.

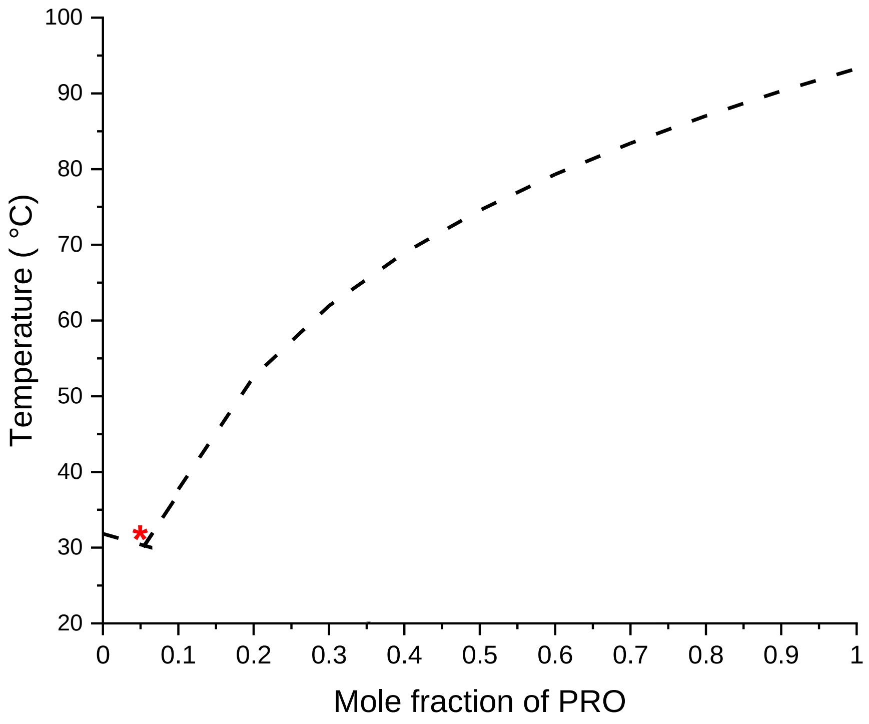

Figure S11. Theoretical prediction of temperature and composition of the eutectic formed between PRO and CAP. The theoretical liquidus curves were calculated using the Schroeder van Laar equation. Using this equation, it was found that the calculated eutectic melting temperature is around 30.5 °C and this eutectic phase would comprise 0.055 mol of PRO.

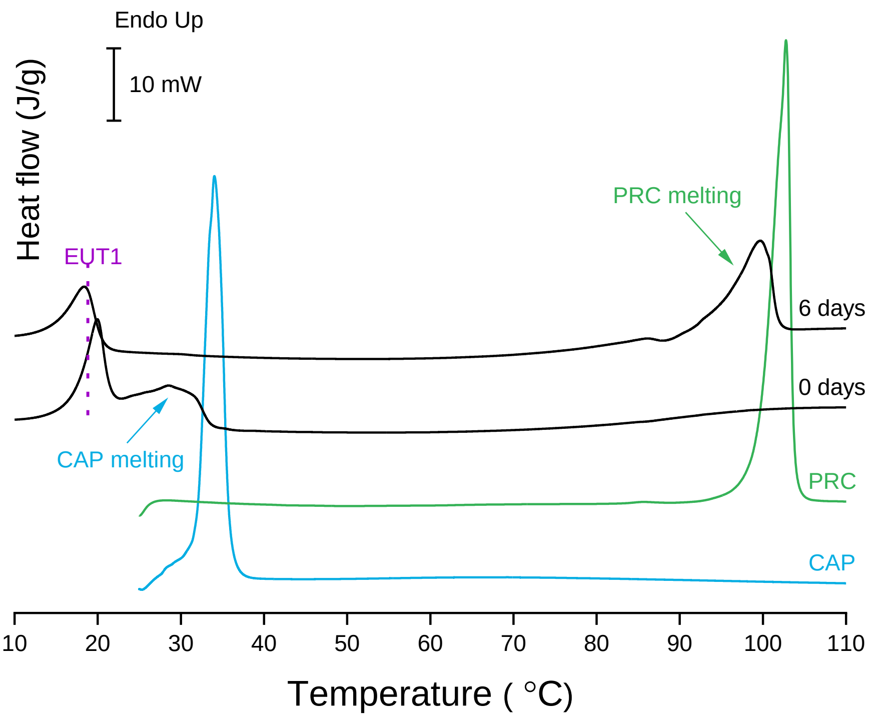

Figure S12. DSC thermograms of CAP (analysed as received), PRC (crystallised from ethanol) and samples prepared by co-grinding PRO and CAP in an agate mortar at 0.2 mol fraction of PRO at 4 °C.

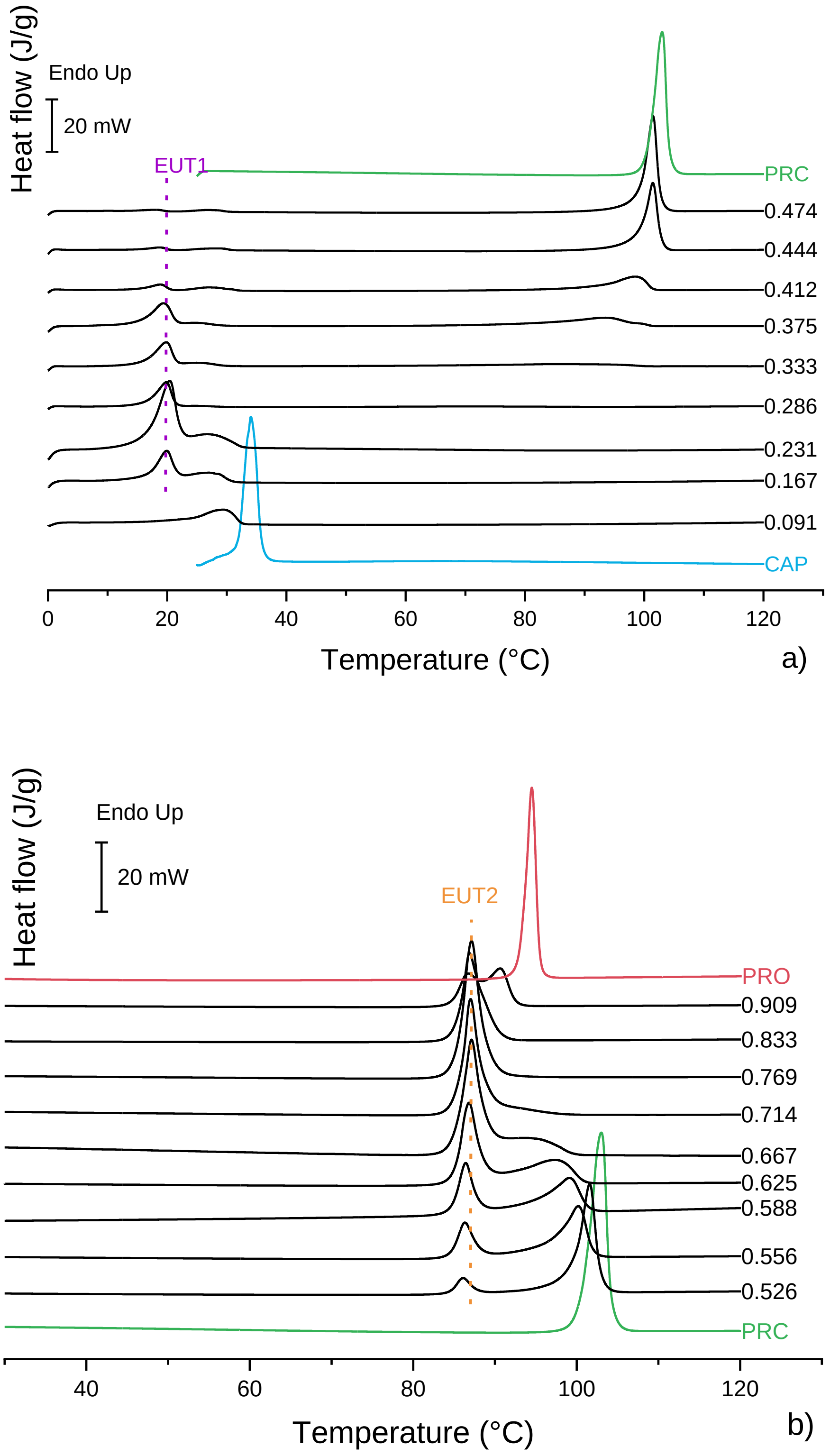
Heat flow (J/g)
Endo Up
20 mW
EUT1
PRC
0.474
0.444
0.412
0.375
0.333
0.286
0.231
0.167
0.091
CAP
0
20
40
60
80
100
120
Temperature (°C)
a)
Heat flow (J/g)
Endo Up
20 mW
EUT2
PRO
0.909
0.833
0.769
0.714
0.667
0.625
0.588
0.556
0.526
PRC
40
60
80
100
120
Temperature (°C)
b)

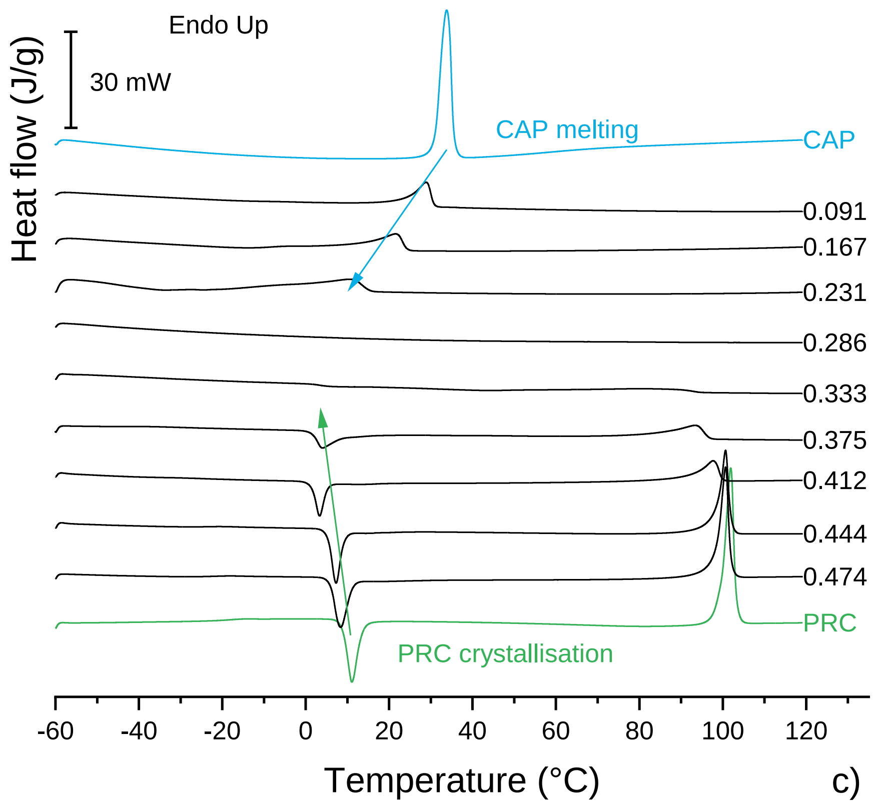

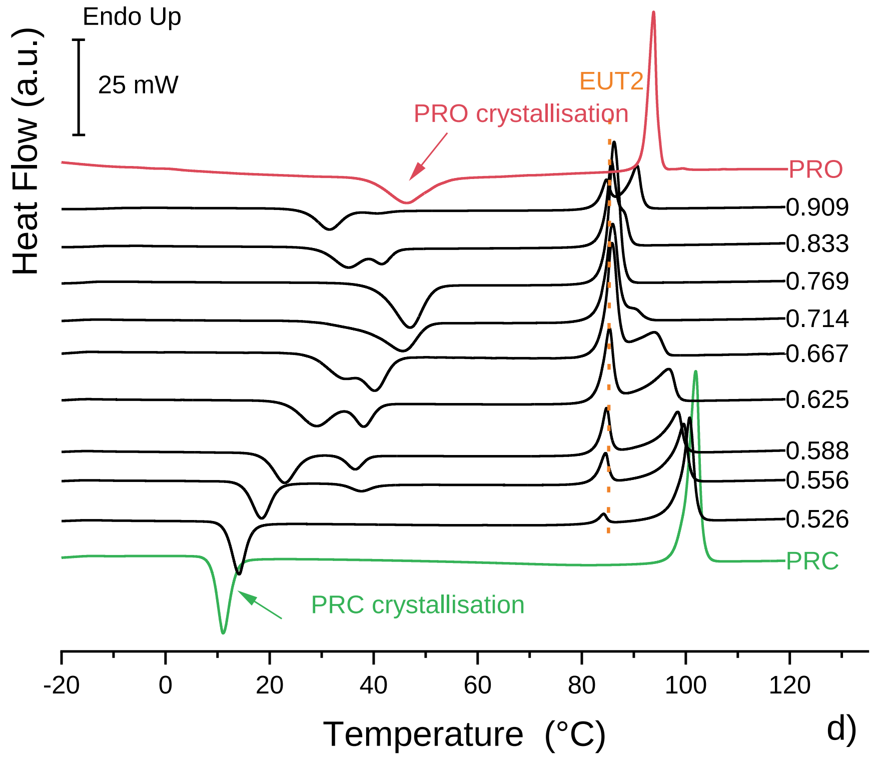

Figure S13. a) DSC traces of the CAP-PRC physical mixtures from the first heating cycle; b) DSC traces of the PRO-PRC physical mixtures from the first heating cycle; c) DSC 2nd heating cycle thermograms of CAP-PRC systems and d) DSC 2nd heating cycle thermograms of PRO-PRC systems. The labels represent the mol fraction of PRO.

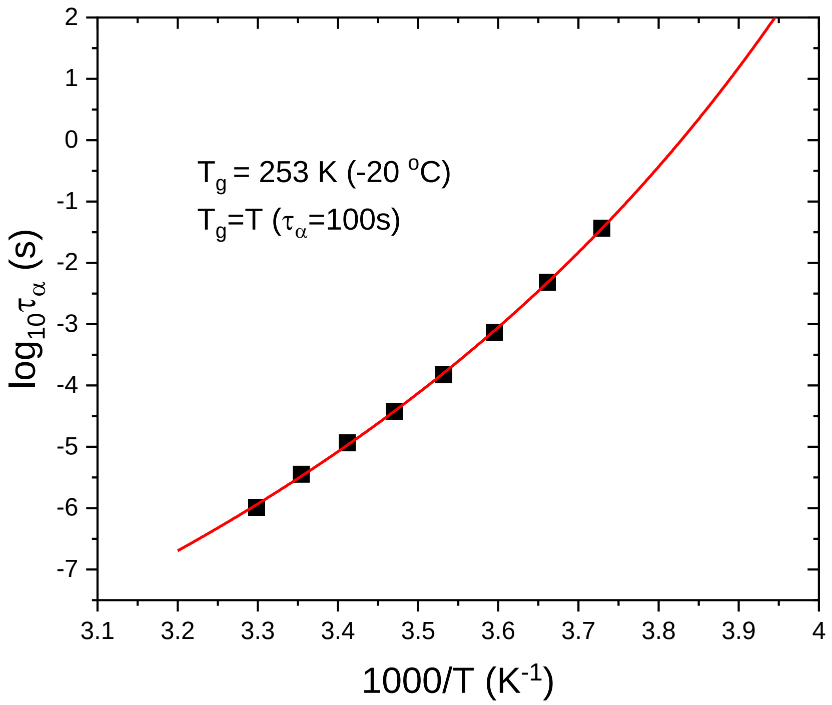

Figure S14. Relaxation map of EUT2. The solid line denotes VFT fit of experimental data.

References:

1. Stott, P.W.; Williams, A.C.; Barry, B.W. Mechanistic study into the enhanced transdermal permeation of a model β-blocker, propranolol, by fatty acids: A melting point depression effect. Int. J. Pharm. 2001, 219, 161–176, doi:10.1016/S0378-5173(01)00645-7.
